# Statistical multi-level shape models for scalable modeling of multi-organ anatomies

**DOI:** 10.3389/fbioe.2023.1089113

**Published:** 2023-02-16

**Authors:** Nawazish Khan, Andrew C. Peterson, Benjamin Aubert, Alan Morris, Penny R. Atkins, Amy L. Lenz, Andrew E. Anderson, Shireen Y. Elhabian

**Affiliations:** ^1^ Scientific Computing and Imaging Institute, University of Utah, Salt Lake City, UT, United States; ^2^ School of Computing, University of Utah, Salt Lake City, UT, United States; ^3^ Department of Orthopaedics, School of Medicine, University of Utah, Salt Lake City, UT, United States; ^4^ EOS Imaging Inc., Montreal, QC, Canada

**Keywords:** computational anatomy, hierarchical statistical models, statistical shape modeling, computational morphometrics, shape and relative pose models, vertebra, foot and ankle, hip and pelvis

## Abstract

Statistical shape modeling is an indispensable tool in the quantitative analysis of anatomies. Particle-based shape modeling (PSM) is a state-of-the-art approach that enables the learning of population-level shape representation from medical imaging data (e.g., CT, MRI) and the associated 3D models of anatomy generated from them. PSM optimizes the placement of a dense set of landmarks (i.e., correspondence points) on a given shape cohort. PSM supports multi-organ modeling as a particular case of the conventional single-organ framework *via* a global statistical model, where multi-structure anatomy is considered as a single structure. However, global multi-organ models are not scalable for many organs, induce anatomical inconsistencies, and result in entangled shape statistics where modes of shape variation reflect both within- and between-organ variations. Hence, there is a need for an efficient modeling approach that can capture the inter-organ relations (i.e., pose variations) of the complex anatomy while simultaneously optimizing the morphological changes of each organ and capturing the population-level statistics. This paper leverages the PSM approach and proposes a new approach for correspondence-point optimization of multiple organs that overcomes these limitations. The central idea of multilevel component analysis, is that the shape statistics consists of two mutually orthogonal subspaces: the within-organ subspace and the between-organ subspace. We formulate the correspondence optimization objective using this generative model. We evaluate the proposed method using synthetic shape data and clinical data for articulated joint structures of the spine, foot and ankle, and hip joint.

## 1 Introduction

Human anatomy is spatially and hierarchically organized into complex, interrelated, and interacting organs with definite shapes (i.e., forms) tied to their function. These shapes vary substantially across populations Cerrolaza et al. (2019). Form and function can also adapt in response to many biological processes, including morphogenesis, injury, disease, and death Costafreda et al. (2011), Lindberg et al. (2012), Carriere et al. (2014), Zhang et al. (2009), and Sciancalepore et al. (2012). Statistical shape modeling (SSM) is an enabling quantitative tool in medical and biological sciences to study form and function. SSM parses the anatomy into a quantitative representation that facilitates testing of biologically relevant hypotheses by defining an *anatomical mapping across a population* of 3D models of anatomy generated from medical imaging data (e.g., CT, MRI). Studying multiple organs together, especially for complex anatomical structures like that of the subcortical brain or articulated joints, can reveal crucial insights, which can help explore links between changes in anatomy due to pathology and the underlying biological process. Thus, most clinical applications encourage statistical shape modeling of multiple organs together instead of single organ structures outside their multi-organ context Gorczowski et al. (2007). Unlike a single-organ model, a multi-organ shape model should capture both organ-specific variability and inter-organ relations to accurately represent complex anatomies and derive quantitative metrics on mechanisms and progression of biological processes. Inter-organ relations can also provide contextual information for expert-driven and automated interpretation of medical images in applications such as radiotherapy planning, diagnosis, and treatment planning Fritscher et al. (2014) and Si and Heng (2017). Furthermore, multi-organ models can advantageously introduce statistical priors for complex periodic multi-structures, such as the spine, to apply non-rigid or poly-rigid image registration in intraoperative guidance imaging Drobny et al. (2020). However, inter-organ relations are either user-defined (e.g., Cerrolaza et al. (2013) and Cerrolaza et al. (2011), limited by their generality and practicality for an arbitrary number of organs, or usually estimated in isolation by learning intra-organ variability, resulting in sub-optimal models Cerrolaza et al. (2019). Moreover, hierarchical models such as Lecron et al. (2012) rely on pre-built shape models that were constructed independently, for further statistical analyses, sacrificing anatomical integrity and inducing anatomical inconsistencies Cerrolaza et al. (2019).

Anatomical mappings can be represented implicitly using deformation fields or explicitly using a set of landmarks (or point correspondences) that are defined consistently across the population. Implicit representations hold promise, but finding the transformation that quantifies differences among shapes is challenging. Explicit representations, which are the focus of this work, provide more interpretable results for statistical analyses and visualization Zachow (2015). These mappings should be learned from the study population in a data-driven manner to capture the underlying population-specific morphological variability Kulis et al. (2013). Approaches for establishing such mappings that rely on pairwise comparisons (e.g., Styner et al. (2006) and Jenkinson et al. (2012)] typically require a predefined atlas for initialization, leading to biased and suboptimal models Goparaju et al. (2022). Group-wise approaches [e.g., Durrleman et al. (2014) and Cates et al. (2017a)], on the other hand, observe the entire population to quantify the quality of shape correspondences, and hence better reflect the underlying population variability Goparaju et al. (2022). Particle-based shape modeling (PSM) Cates et al. (2007) and Cates et al. (2017a), in particular, is a state-of-the-art computational approach for constructing point distribution models (PDM) *via* automatically placing a dense set of corresponding landmarks on a set of shapes. The scientific and clinical utility of PSM have been demonstrated in image and shape analysis [e.g., Bhalodia et al. (2021) and Shigwan et al. (2020)], neuroscience [e.g., Sultana et al. (2019) and Audette et al. (2017)], biological phenotyping [e.g., Jones et al. (2013) and Cates et al. (2017b)], cardiology [e.g., Bieging et al. (2018) and Goparaju et al. (2022)], and orthopaedics [e.g., Lenz et al. (2021), Goparaju et al. (2022), Krähenbühl et al. (2020), Jacxsens et al. (2020), Atkins et al. (2017a), Atkins et al. (2019), Atkins et al. (2017b), and Atkins et al. (2022)].

PSM supports multi-organ modeling using a global statistical model, similar to other landmarks-based models [e.g., Picazo et al. (2018), Kokko et al. (2021), and Li et al. (2016)]. This is due to its computational simplicity and benefits over single-organ models Wilms et al. (2017). In this modeling scheme, the multi-structure anatomy is considered a single structure and landmarks positions are optimized in the full shared shape space Cates et al. (2008) and Agrawal et al. (2020). However, global multi-organ models suffer from anatomical inconsistencies (e.g., overlapping neighboring organs) and make subtle morphological differences within each organ less obvious Cerrolaza et al. (2019). Global shape models are not computationally and statistically scalable to an arbitrarily large number of organs, with each represented by many landmarks to describe their shapes accurately. Thus, substantially large sample sizes are indispensable for global shape models to have sufficient statistical power and this being reinforced by higher dimensionality of the number of landmarks, leads to a significant memory footprint for correlations computation Cerrolaza et al. (2019) and Jung and Marron (2009). Furthermore, optimizing in the shared shape space of multi-organ structures does not separate shape from pose variations and entangles both intra- and inter-organ modes of variation, making the interpretation of the articulated shape models challenging to relate to clinically relevant insights necessary for the diagnosis of joint misalignment, pathological deformity, and bone abnormalities. On the other hand, the individual modeling approach for multi-organ complexes independently builds the statistical model of each organ. These models fail to capture the inter-organ anatomical patterns completely Yao et al. (2016).

In this paper, we propose a multi-level statistical shape modeling approach that overcomes the limitations of the global shape modeling scheme. We disentangle the shared shape space used in the global shape modeling technique into within-organ and between-organs subspaces to model the intra-organ shape and inter-organ pose variabilities. We formulate the training objective to optimize the point correspondences across the ensemble of multi-organ anatomies in the disentangled shape space, which makes it easily scalable to model multiple organs together without generating anatomical inconsistencies. We demonstrate that the Point Distribution Model (PDM) generated from the proposed shape modeling technique effectively captures the shape variation of each organ while simultaneously reflecting the relative pose variations between the organs in the shape complex. We use synthetic data for proof of concept and real clinical data with downstream validation tasks to demonstrate the efficacy of the proposed method for articulated structures. The proposed shape modeling technique is referred to as the Multi-Level Multi-Organ (MLMO) shape modeling technique.

## 2 Methodology

### 2.1 Background—Particle-based shape modeling for single-organ anatomy

We define the shape surface for an organ structure as a smooth manifold of codimension one, which is a subset of $R^{d}$ . We have *d* = 3 in this work as the shapes are segmented from 3D volumetric images. The particle-based shape modeling (PSM) approach optimizes population-specific shape representations by sampling each surface in a consistently ordered fashion. Each surface $S\subset R^{d}$ can be sampled using a discrete set of *M* points ${\left\{x^{m}\in R^{d}\right\}}_{m=1}^{M}$ that define the configuration space to capture the geometry for each sample. The particle positions **z** = (**x** ^1^, **x** ^2^, … **x** ^*M*^) are the realizations of the random variable **X** for configuration space associated with its probability density function *p* (**X** = **x**). Consider an ensemble E that consists of shape surfaces defined for *N* subjects as $E=\left\{z_{1},z_{2},\ldots ,z_{N}\right\}$ such that each surface has its own set of particles after factoring out global transformations that are irrelevant to modeling shape variations. This defines the shape space such that the vector of *M* particle positions for each surface in the configuration space is mapped to a single point in *dM* − dimensional shape space. Each surface **z**_*n*_ of the ensemble is an instance of the shape space random variable **Z** associated with its probability density function *p* (**Z** = **z**). PSM assumes that the shape space is modeled by a Gaussian distribution as **Z** ∼ (***μ***, **Σ**). Correspondences across the ensemble are established by minimizing an entropy-based objective function that is a combined cost function Q for shape correspondence and surface sampling defined as:

$$Q=H\left(Z\right)-\overset{N}{\underset{n=1}{\sum }}H\left(X_{n}\right)$$ (1)

where *H* (.) denotes the estimation of the entropy function. The differential entropy of *p*(**X**) is given as

$$H\left(X\right)=-\int_{E}p\left(X\right)\log p\left(X\right)dx=-E\left\{\log p\left(X\right)\right\}\approx -\frac{1}{M}\overset{M}{\underset{m=1}{\sum }}\log p\left(x_{m}\right)$$ (2)

The cost function *Q* is minimized using a gradient descent algorithm. The first term in Eq. 1 encourages a compact distribution of the samples in the shape space such that particles are in good correspondence across the shapes. The second term favors uniformly-distributed correspondence positions on the shape surfaces to accurately capture the geometric details of the shape. For a stable optimization of these terms, shape statistics ***μ*** and **Σ** are allowed to lag when particle positions are updated and the negative gradient update $-\frac{\partial H\left(Z\right)}{\partial Z}$ in the shape space is computed once per optimization iteration. The individual shape-based updates in configuration space $\frac{\partial H\left(X_{n}\right)}{\partial X_{n}}$ are then combined to provide the update for each particle. More details related to the optimization technique and gradient updates can be found in Cates et al. (2007) and Cates et al. (2017a).

### 2.2 Multi-organ shape modeling–Problem formulation

A multi-organ (or multi-object) shape complex is defined as a set of solid shapes, each representing a single and connected biological structure, assembled together within a common coordinate frame. This shape complex contains the shape, scale, and positional information for each organ structure, thereby containing the relative pose and orientation between different organ structures in the shape complex. Multi-organ shape structures have alignment variations between the organs that reflect subject-wise anatomical variations relevant to how the organs are relatively positioned and aligned with respect to each other. These alignment variations should not be factored out by the initial rigid alignment techniques that are usually performed prior to the shape modeling process. These geometric relationships between the organs are of significant importance, especially in biomechanics-based shape modeling Agrawal et al. (2020), Zhang et al. (2016), and Kainmueller et al. (2009).

Here, we define the notations for the multi-organ shape modeling problem that will be used in the following sections. Given an ensemble E of *N* subjects such that each subject has 3D surfaces defined for *K* organs. Thus, the ensemble is defined as $E={\left\{{\left\{z_{n,k}\right\}}_{k=1}^{K}\right\}}_{n=1}^{N}$ . Each surface (or shape) is represented by a set of *M*_*k*_ correspondence particles, where each particle is *d* − dimensional^1^ such that $M=\sum_{k=1}^{K}M_{k}$ is the total number of particles representing a multi-organ shape sample. **x**_*n*,*k*_ is the realization of the configuration space random variable **X**_*n*,*k*_ for the *n* − th subject and *k* − th organ and the corresponding shape space variable is **Z**_*n*,*k*_ such that its realization is $z_{n,k}=\left[x_{n,k}^{1},x_{n,k}^{2},\ldots ,x_{n,k}^{M_{k}}\right]\in R^{dM_{k}}$ .

### 2.3 Global shape modeling for multi-object complexes

To capture shape statistics in multi-organ anatomies, Cates et al. (2008) extended the concept of particle-based shape modeling for single objects as described in Section 2.1, and presented an optimization scheme where multiple organs are treated as a single structure. Here, the shape space variable $Z\in R^{dM}$ is the concatenation of the random variables defined for each organ $Z_{n,k}\in R^{dM_{k}}$ . The optimization objective here is the combined ensemble and shape cost function which is defined as:

$$Q=\alpha H\left(Z\right)-\overset{K}{\underset{k=1}{\sum }}\overset{N}{\underset{n=1}{\sum }}H\left(X_{n,k}\right)$$ (3)

where *H* is the differential entropy of the corresponding random variable and *α* is the relative weighting parameter. The first term in Eq. 3 represents the ensemble entropy in shape space **Z** and minimizing this produces a compact representation of the model, and hence lowers the complexity of the shape model. The second term in Eq. 3 represents the surface entropy in the configuration space **X**, which on maximizing gives a uniform distribution of correspondence particles across shape surfaces. This formulation assumes that object-level correspondence of each organ is known a priori. This formulation decouples the spatial interaction between the particles on different organs by constraining each particle to stay on the shape surface of a single organ, but the ensemble entropy is minimized in a shared shape space by modeling the entire multi-organ shape sample $z\in R^{dM}$ as an instance of the random variable **Z** that is assumed to be Gaussian-distributed and follows a generative model described as,

$$z=\mu +\epsilon ,\epsilon ∼N\left(0,\Sigma \right)$$ (4)

The covariance matrix **Σ** includes all particle positions across the entire multi-organ shape, forcing the optimization to take place in the shared shape space of all organs. Minimizing the entropy of this distribution favors high spatial correlations between corresponding samples of the entire multi-organ shape complex across the population without incorporating how these organs interact with each other across the population. Treating the multi-organ complex as a single object, leads to the oversimplification of the complex human anatomy and fails to capture the variabilities within the organ and the interactions between the organ. To produce a compact statistical representation of the shape complex as a whole, the global shape modeling technique might not capture the individual morphological changes of each organ and their interactions correctly by placing correspondence particles that are anatomically inconsistent.

### 2.4 Multi-level component analysis

One of the widely used approaches to characterize the variability of shapes represented by a point distribution model (PDM) is the Principal Component Analysis (PCA) that allow both visualization and dimensionality reduction. The basis vectors defined by PCA are optimal in the least squares sense as each basis vector is chosen to minimize the sum-of-squares (SSQ) residual error in data. The basis vectors describe the independent modes of variation by accounting for the correlations among the correspondence positions of the particles.

The Multilevel Component Analysis (MLCA) Timmerman (2006), an extension of PCA, is used to analyze hierarchical structures in multi-object models. More specifically, the correspondence particle $z_{n,k}^{m}\in R^{d}$ is observed at two levels—a local within level, where shape variation is identified in each individual organ, and at a global level, where the relative pose of each organ is observed in the multi-organ shape complex. Using this model, the generative model of a particle can be formulated as follows:

$$z_{n,k}^{m}=\underset{\text{offset}}{\underbrace{{\bar{z}}_{n}}}+\underset{\text{within}-\text{organ}}{\underbrace{\left(z_{n,k}^{m}-{\bar{z}}_{n,k}\right)}}+\underset{\text{between}-\text{organs}}{\underbrace{\left({\bar{z}}_{n,k}-{\bar{z}}_{n}\right)}},$$ (5)

where ${\bar{z}}_{n}=\frac{1}{M}\sum_{k=1}^{K}\sum_{m=1}^{M_{k}}z_{n,k}^{m}$ is the offset term representing the global centroid of the multi-organ shape complex and ${\bar{z}}_{n,k}=\frac{1}{M_{k}}\sum_{m=1}^{M_{k}}z_{n,k}^{m}$ is the centroid of the *k* − th organ. The second term of Eq. 5 encodes within shape organ variations, which is the deviation of the correspondence particle of each organ from its own centroid. The last term of Eq. 5 encodes the between organs pose variations, which is the relative pose changes of each organ in the multi-organ shape complex from the global centroid of the shape complex.

MLCA uses the notion of Analysis of Variance (ANOVA) to split the total sum of squares into components that are related to the effects used in the model. For Eq.5, we can write SSQ residual errors as:

$$\underset{ssq_{\mathrm{total}}}{\underbrace{\underset{m,n,k}{\sum }{\left(z_{n,k}^{m}\right)}^{2}}}=\underset{ssq_{\mathrm{within}}}{\underbrace{\underset{m,n,k}{\sum }{\left(z_{n,k}^{m}-{\bar{z}}_{n,k}\right)}^{2}}}+\underset{ssq_{\mathrm{between}}}{\underbrace{\underset{n,k}{\sum }{\left({\bar{z}}_{n,k}-{\bar{z}}_{n}\right)}^{2}}}$$ (6)

In Component Analysis (CA) models such as PCA, the main goal is to approximate the data in the best possible manner in the least-squares sense. By Eq. 6, the total sum of squares for correspondence particle data can be split into two levels—within and between, then it is natural to explain the best possible sum of squares at each level by building a two-level component model. Thus, MLCA gives a general formulation of such a two-level component model. Here, we build the component model at each level—in the within subspace for each organ and in the between subspace for all the organs together. This gives us *K* + 1 mutually orthogonal subspaces and we assume each of these subspaces can be modeled by a Gaussian distribution. Thus, analogous to PCA, the shape vector describing each organ $z_{k}\in R^{dM_{k}}$ can be expressed by a linear combination of the basis vectors of the within subspace and between subspace as follows:

$$z_{k}=\mu +U_{k}^{W}\alpha_{k}^{W}+U_{k}^{B}\alpha_{k}^{B}$$ (7)

where $\mu \in R^{dM_{k}}$ is the consolidated mean of the within and between subspace and the offset, $U_{k}^{W}\in R^{dM_{k}\times N}$ is the matrix of principal components of the within subspace of organ *k* and $U_{k}^{B}\in R^{dM_{k}\times N}$ is the sub-matrix of principal components of between subspace that belongs to organ *k*. The coefficient vectors $\alpha_{k}^{W}\in R^{N}$ of the within subspace are distributed according to $N\left(0,\Sigma_{k}^{W}\right)$ and the coefficient vectors $\alpha^{B}\in R^{N}$ are distributed according to $N\left(0,\Sigma^{B}\right)$ where Σ* denotes the covariance matrix of the within subspaces of each organ and the between subspace defined respectively by the within and between terms of Eq. 5. This formulation of MLCA, gives us an analysis technique by which we can probe the configuration variations of articulated joints or other multi-organ anatomies separately from the morphological or shape variations.

### 2.5 Proposed shape modeling approach—multi-level multi-organ shape modeling

We propose a novel optimization scheme for multi-organ shape complexes that disentangles the shared shape space of the multiple organs into relevant subspaces. We build our hypothesis from the generative model in Eq. 7 of multi-level component analysis. The shared shape space for the multi-organ structure can be split into individual subspaces for each organ, which models the shape variability within each organ, and a common subspace, which accounts for the relative pose variability between the organs in the shape sample. The random variable **Z** is replaced by a sequence of random variables $Z_{k}^{W}$ , which models the shape variability of each organ, and another random variable **Z** ^*B*^ for the interactions between each organ across the population. The shape morphology variations for each organ *k* and their relative alignment in the multi-organ complex are encoded in the within vectors for each organ $z_{k}^{W}$ and a between vector **z** ^*B*^ by splitting the particles in the shape vector **z** in terms of the deviation of the centroid of each organ and centroid of the multi-organ complex according to within and between terms of Eq. 5. We propose a new cost function that minimizes the entropy of each individual within subspaces and between subspace as follows.

$$Q=\alpha_{W}\overset{K}{\underset{k=1}{\sum }}H\left(Z_{k}^{W}\right)+\alpha_{B}H\left(Z^{B}\right)-\overset{K}{\underset{k=1}{\sum }}\overset{N}{\underset{n=1}{\sum }}H\left(X_{n,k}\right)$$ (8)

where *H* is the differential entropy function; *α*_*W*_ and *α*_*B*_ are the relative weighting parameters for the within and between subspaces, respectively, that define the contribution of the correspondence objective of that subspace to the particle optimization process. The within subspaces for each organ and the between subspace are modeled as Gaussian distributions $p\left(Z_{k}^{W}\right)$ and *p* (**Z** ^*B*^) with covariances $\Sigma_{k}^{W}$ and **Σ** ^*B*^, respectively. We estimate these covariance matrices directly from the data using the within and between parts of Eq. 5. The objective function *Q* in the above Eq. 8 is minimized in such a way that correspondence particle updates for the within and between subspaces are made in an alternating fashion. We first make particle updates by computing the gradient in the within subspace for each organ and then make changes in the relative alignment in each organ by computing the gradient updates in the between subspace. In this way, we disentangle the shape from pose in the optimization process, while simultaneously preserving the anatomical correctness of the articulation of the joint. The entropy terms for the within and between subspace in Eq. 8 are given by:

$$H\left(Z_{k}^{W}\right)\approx \frac{1}{2}\log\Sigma_{k}^{W}=\frac{1}{2}\overset{dM_{k}}{\underset{i=1}{\sum }}\log\lambda_{k,i}^{W}$$ (9)

$$H\left(Z^{B}\right)\approx \frac{1}{2}\log\Sigma^{B}=\frac{1}{2}\overset{dK}{\underset{i=1}{\sum }}\log\lambda_{i}^{B}$$ (10)

where $\lambda_{k,i}^{W}$ and $\lambda_{i}^{B}$ denotes the eigenvalues of $\Sigma_{k}^{W}$ and **Σ** ^*B*^ respectively. The gradient updates of each subspace is computed as:

$$\frac{-\partial H\left(Z^{⋆}\right)}{\partial X}\approx Y^{⋆}{\left(Y^{⋆⊤}Y^{⋆}+\alpha I\right)}^{-1}$$ (11)

where **Z** ^⋆^ denotes the subspace and **Y** ^⋆^ denotes the mean centered matrix of that subspace. Here, ⋆ can be *W* or *B* representing the within and between subspace, respectively. The dimensionality of the particle correlation matrices in these disentangled within and between subspaces $\Sigma_{k}^{W}$ and **Σ** ^*B*^ are *dM*_*k*_ × *dM*_*k*_ and *dK* × *dK*, respectively, which is much lower than the dimensionality of the correlation matrix in global shape modeling approach which is *dM* × *dM*. This disentangled formulation in the proposed MLMO method gives relief in computational burden as it leads to faster eigenvalue decomposition of the correlation matrix of significantly lower dimension used for entropy computations (Eqs. 9, 10) and consequently, faster optimization as compared to the global shape modeling approach. Moreover, this makes the MLMO model more flexible and less constrained, demonstrating its better statistical power under high dimensional and low sample-size settings which is more predominant in multi-object shape modeling scenarios.

### 2.6 Evaluation metrics

In this section, we describe different quantitative and qualitative metrics used to systematically evaluate the results produced by the underlying PSM method and the associated shape correspondence performance.

#### 2.6.1 Qualitative metrics

We use mean and modes of variation to qualitatively assess the shape model. PCA is a linear transformation of data into new coordinate space, in which each coordinate axis represents decreasing amount of variability in the data. In MLCA, this transformation is done at different levels in which the data is observed. The point correspondences generated by the respective PSM technique is subjected to PCA in the shared subspace and MLCA in the within-organ and between-organs subspaces. This provides a ranking of the uncorrelated modes of variation based on the amount of variance explained relative to the total variance. When PCA is performed in the shared shape space, the modes of variation for the morphology and relative pose of the multi-organ shape structure remain entangled. This limits the ability of the shape model to discover hidden patterns in the shape class of interest that can be clinically relevant. MLCA disentangles shape morphology and alignment variations in multi-organ shape complexes. This helps in factoring out significant variations of how the shape morphology of each organ changes across the population and also how the relative alignment of multi-organ shape varies across the population. We visualize and describe these qualitative modes for the clinical data in Section 3 by examining the anatomical correctness and integrity of the mean shape and its associated modes.

#### 2.6.2 Quantitative evaluation metrics

We use the quantitative metrics of compactness, generalization, and specificity Davies (2002) to assess the shape-correspondence performances with respect to the PDM construction. These measures are defined under the assumption that the shape model is inherently built using a PCA generative process. We extend these metrics for MLCA by defining these evaluation measures for within-organ and between-organs subspaces for the multi-organ shape model. These measures collectively quantify the quality of the shape model constructed from correspondence particles and are defined as a function of number of modes *P* under consideration.

##### 2.6.2.1 Compactness

Multi-organ shape models inherently have high dimensionality but this high dimensional shape space can be parameterized by a low-dimensional subspace (shared, within and between) in terms of eigenvectors and associated eigenvalues. *Compactness* is the evaluation metric that quantifies the amount of variance of the underlying shape model. For a given subspace *S*, compactness is computed as sum of the eigenvalues $\lambda_{p}^{S}$ up to the *P* − th mode as $C\left(P\right)=\sum_{p=1}^{P}\lambda_{p}^{S}$ , where *S* can be *G*, *W* or *B* denoting the global shared, within or between subspaces, respectively. A shape model with higher compactness measure is better as it can explain the shape and/or pose variability with fewer modes of variation.

##### 2.6.2.2 Generalization

*Generalization* is defined as the ability of the shape model to represent unseen shapes of the structure modeled. It is quantified as an approximation error using the leave-one-out cross validation approach where a testing shape vector is left out from *N* shape vectors and the shape model is obtained from the remaining *N* − 1 shape vectors. The approximation error is then calculated in terms of Euclidean distance (in *mm*) between the held-out shape instance and its reconstruction from the shape model. For a multi-organ shape model, we compute this metric in different subspaces—shared, within and between, which quantifies how well the shape model can generalize shape vectors from that subspace. Generalization is thus quantified as $Generalization\left(P\right)=\frac{1}{N}\sum_{n=1}^{N}E_{n}\left(P\right)$ where, $E_{n}\left(P\right)=\|z_{n}^{S}\left(P\right)-z_{n}^{S}{\|}^{2}$ is the approximation error between shape vector $z_{n}^{S}\left(P\right)$ reconstructed using *P* modes, $z_{n}^{S}$ is the held out shape vector, and *S* can be either *G*, *W* or *B* denoting the global shared, within or between subspaces respectively. The shape vector reconstruction for the shared subspace follows the PCA generative Eq. 4 and the reconstruction for the within and between subspace follows the MLCA generative Eq. 7. For two shape models built using the same training data, the model having a lower value of generalization error indicates a more efficient shape model that can better represent unseen shape structures. By computing generalization in these different subspaces, we can assess how well a shape model can learn the characteristics of the multi-organ shape and it’s relative alignment modeled from a limited training set. If the multi-organ shape model is over-fitted to the training data, it will not be able to generalize well to unseen examples and this would be highlighted by a higher generalization error. A lower generalization error in the within subspace denotes that the shape model can easily generalize the morphological changes of a particular organ and similarly a lower generalization error in the between subspace indicates that the shape model can easily generalize the relative pose of the multi-organ shape complex (denoted by the centroid of each organ).

##### 2.6.2.3 Specificity

*Specificity* quantifies the ability of the shape model to generate new plausible instances of the shapes by constraining the variability in the shape space using the learned population-specific shape statistics. To compute this metric, we randomly sample a large number of vectors (1,000 in our experiments) from the subspace and then compute the approximation error (Euclidean distance, in *mm*) between the randomly sampled shape vector and nearest training sample. Specificity is defined as a function of number of modes *P* considered and computed as $Specificity\left(P\right)=\frac{1}{T}\sum_{t=1}^{T}\|z_{t}^{S}\left(P\right)-z_{t}^{S}{\|}^{2}$ , where *T* is the large number of shape vectors randomly generated, $z_{t}^{S}\left(P\right)$ is the shape vector randomly sampled from the subspace, $z_{t}^{S}$ is the closest training sample and *S* can be either *G*, *W* or *B* denoting shared, within or between subspaces respectively. The randomly sampled vectors for shared subspace are generated using the PCA generative Eq. 4 and the randomly sampled vectors for the within and between subspace are generated using the MLCA Eq. 7. For two shape models, a model with lower value of specificity is better and more specific. A lower specificity value in the within subspace denotes that the shape model is more specific to the morphological changes in the shape model and similarly a lower specificity in the between subspace indicates that the shape model is more specific to the relative pose variations of the multi-object shape complex.

## 3 Experiments and results

We used synthetic and real medical data to demonstrate that the proposed shape modeling approach generates optimal shape models for complex multi-organ anatomical structures. We use the global shape modeling approach described in Section 2.3 as a baseline method for comparison. Shape models can also be created by the individual modeling approach (Section 2.1), where shape models are built separately for each organ in the multi-organ structure. If we model each organ separately, we sacrifice the inter-structural shape and pose correlations, which are of significant interest in many clinical contexts. The main emphasis of this paper is to efficiently bring in these inter-organ relations in the shape model for multi-organ structure. Therefore, in this work, we restrict our comparison only to the joint shape modeling approach. We employed ShapeWorks Cates et al. (2017a) and Cates et al. (2007), an open-source software implementation of the particle-based shape modeling method to build shape models for the clinical and synthetic data. We implemented the baseline method of global shape modeling approach using the optimization scheme already given by ShapeWorks. We modified the objective function to implement our proposed optimization scheme described in Section 2.5. ShapeWorks utilizes an iterative, particle-splitting strategy, in which the full set of particles is initialized in a multiscale fashion such that in every step each particle is split to produce a new nearby particle until the desired number of particles is attained. This mechanism is a self-tuning system of particles that distribute themselves across the shape surface using repulsive forces to achieve optimal point distributions that cover each surface. The number of particles for a particular anatomical shape surface depends on its size, curvature, and morphological variations such that smooth and small shapes require less number of particles as compared to complex and highly variable shapes. In our experiments on different datasets, the number of particles for given anatomy is chosen empirically by utilizing this coarse-to-fine particle splitting strategy until the resulting particle representation is deemed to capture sufficiently good detail for the given anatomy depending on its size and curvature.

### 3.1 Proof of concept experiment

To illustrate and assess the proposed MLMO shape modeling approach, we devised a proof-of-concept experiment using synthetic shapes. We created synthetic data simulating multi-organ structures using supershapes, which are a family of parameterized shapes Gielis (2003). Each object in the multi-object synthetic shape is modeled by a supershape with different number of lobes and shape parameters randomly drawn from a *χ* ^2^ distribution which reflects the morphological changes in each supershape. The relative alignment of supershapes in the multi-object shape complex is modeled by a parabolic curve *y* = *ax* ^2^, where a∼U(0,0.001) for each *x* coordinate of the individual supershape. This results in shape variations in the supershape reflected by the change in one parameter of supershape and the alignment variations of the multi-object shape complex changing from concave to convex. Thus, the synthetic dataset helps us evaluate the shape modeling technique as these shape and pose variations have been incorporated in a known way, and the underlying PSM technique should correctly model these variations.

**FIGURE 1:**
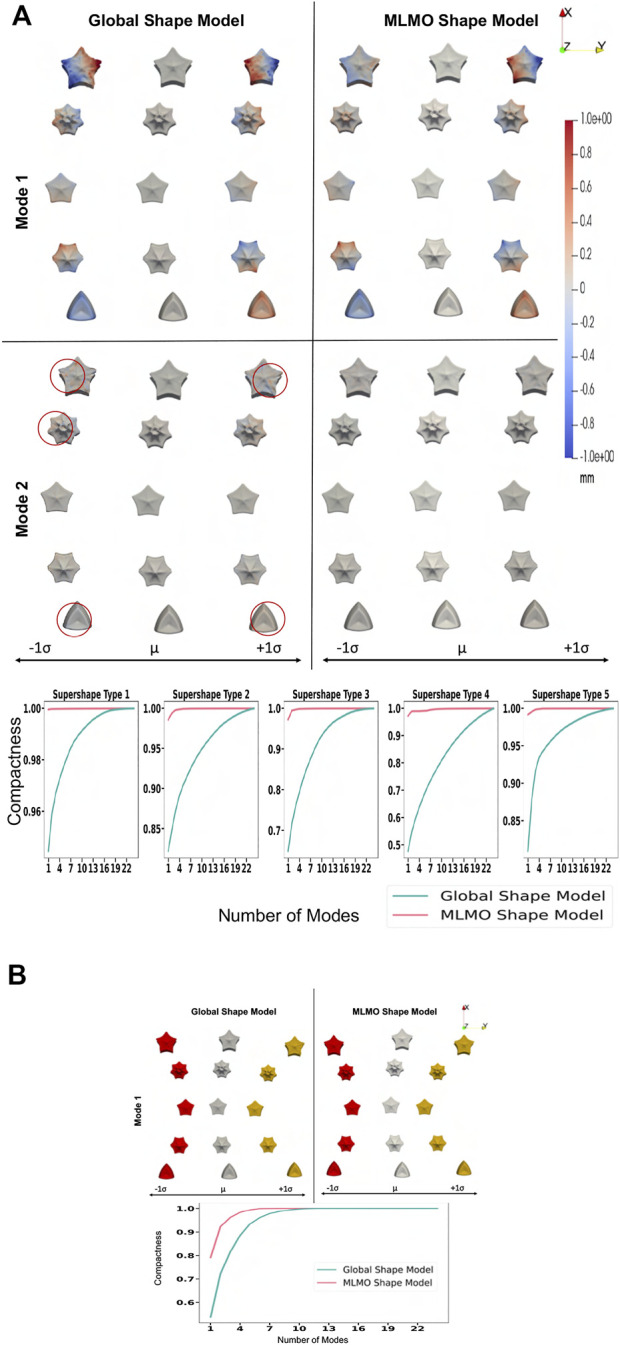
Proof of concept experiment results—Modes of variation and compactness metric. **(A)** Within-object subspace. **(B)** Between-objects subspace.

We build the shape models using 512 correspondence points for each supershape object in the multi-object shape complex. To evaluate the shape model, we use MLCA to visualize the patterns of shape and pose variability across the population. The dominant modes of the shape model should correctly reflect these variabilities, which are known to be only two for the synthetic dataset. Figure 1A shows the within-object morphology variations with the compactness measure for each supershape and Figure 1B shows the between-objects alignment variations and its compactness measure. For the MLMO shape model, there is only one mode of variation in the within-object subspace that shows the shape variability of each supershape and there is only one mode of variation in the between subspace which shows the changes in the alignment of the entire shape structure going from concave to convex. Therefore, it can be concluded that the proposed shape modeling technique correctly disentangled the shape and pose variations and explained the total variability for within and between subspaces with just one mode. On the other hand, although the global shape model correctly identifies the pose variation in the between subspace, it adds shape variations in the secondary within-object modes, which does not truly reflect the shape variations in the actual synthetic dataset. This underpins our hypothesis that optimizing the shape model in the shared shape subspace of a multi-object structure without disentangling the shape from alignment might bring in those variations in morphology or pose that are not anatomically accurate.

### 3.2 Spinal column

#### 3.2.1 Dataset

Publicly available labeled and segmented data for human vertebrae by the vertebrae segmentation challenge (VerSe) Sekuboyina et al. (2021) is used to build shape models. Although this database is large-scale, the number of patients that have the entire spine segmented is limited. A subset of subjects from VerSe is selected such that the number of vertebrae covered in the multi-organ structure of each subject is maximized. 30 patients having complete 17 vertebrae present in the thoracolumbar vertebral region - thoracic (T1 to T12), and lumbar (L1 to L5) were selected. The shape cohort comprises of healthy subjects and the subjects having multiple pathologies related the spinal column, in the age range of 60 ± 17 years. We build the shape model with 8,704 correspondence particles on the whole spine such that 512 particles are placed on each vertebra.

#### 3.2.2 Qualitative results

**FIGURE 2:**
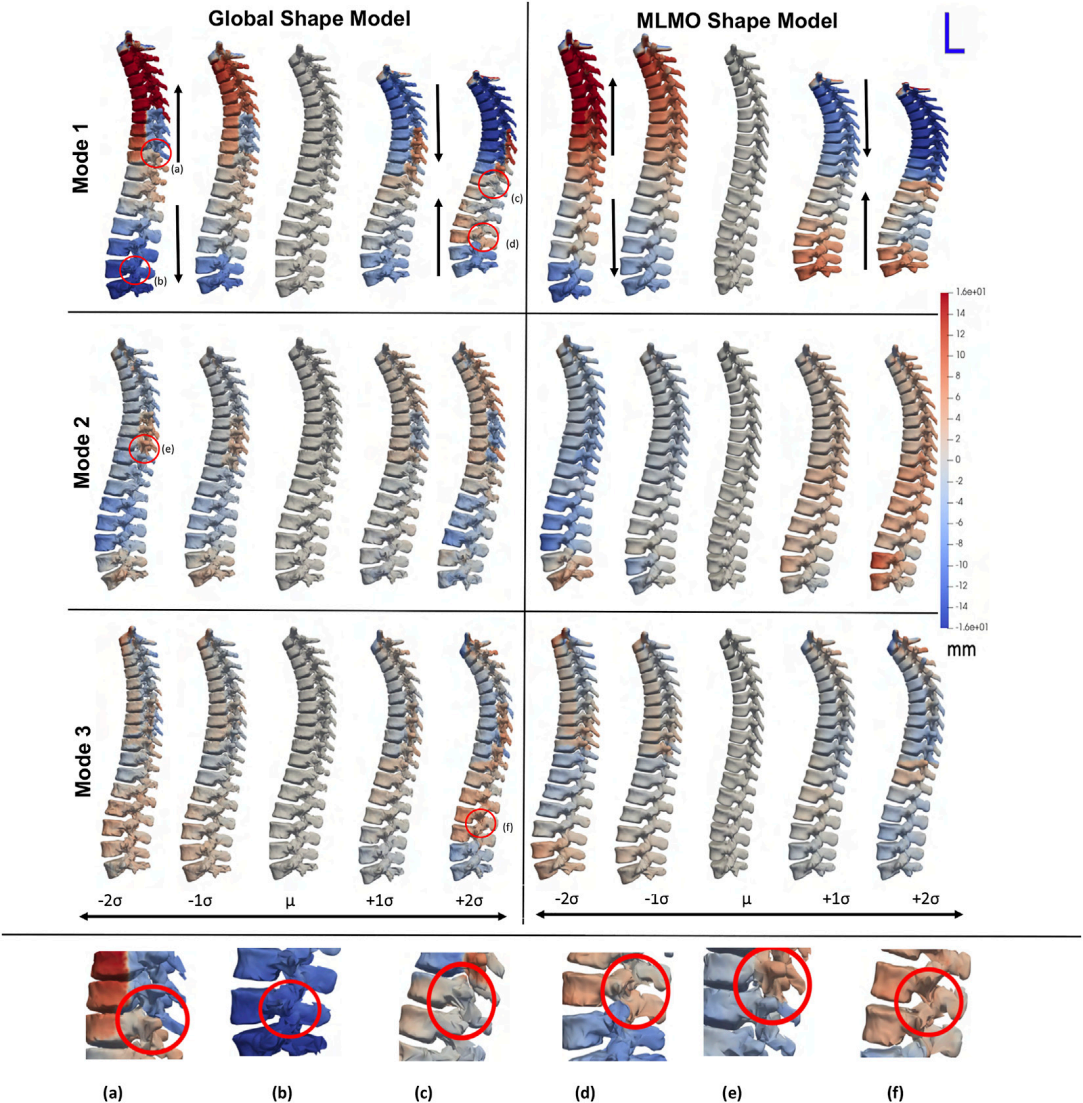
PCA modes of variation computed in the shared subspace for the spinal column dataset. The color map shows the distance of each mode from the mean shape. Some of the anatomical inconsistencies are highlighted in red circles labeled from **(A–F)**.

Figure 2 shows the modes of variation for PCA done in shared shape space. We can see that for both the shape modeling approaches, the modes depicting morphological changes of vertebrae and their relative pose are entangled. The primary mode of variation shows variation in the spinal length with some changes in the morphology of each vertebra. Both pose and local shape changes are inter-twined across these variations. The second and third modes show a variation of kyphosis and lordosis curvatures in the sagittal plane. The particles are in better correspondence with the proposed MLMO shape modeling approach as this is indicated by the smooth reconstructed shapes from the generated particles that are more faithful to the original training shape. The modes of variation for the global shape model show anatomical inconsistencies in regions where the vertebrae come close to each other.

**FIGURE 3:**
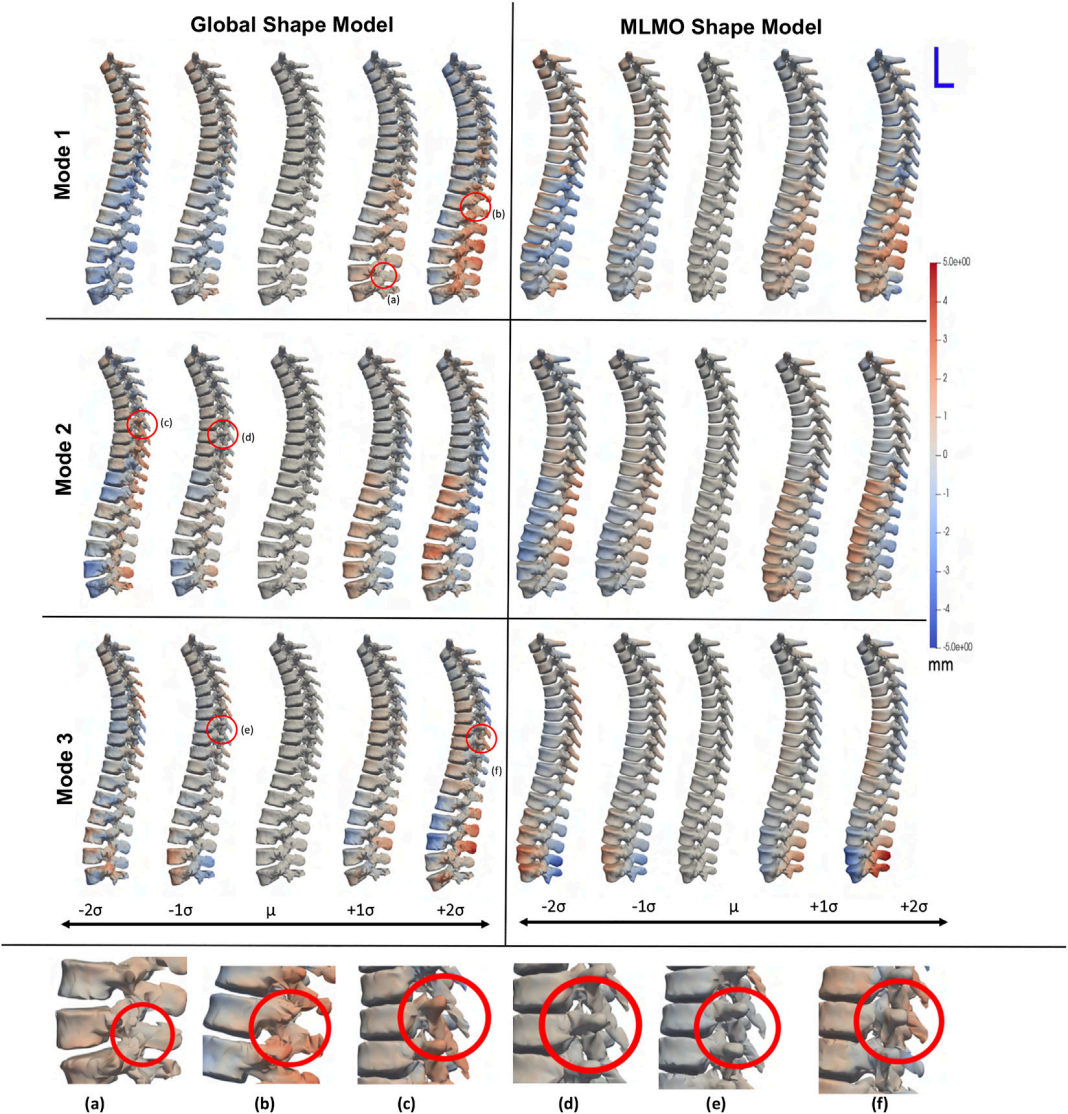
Within-organ modes of variation showing morphological changes in each vertebra. The color map shows the distance of each mode from the mean shape. Some of the anatomical inconsistencies are highlighted in red circles labeled from **(A–F)**.

Figure 3 shows the within-organ modes of variation highlighting only morphological changes in each vertebra. For both shape modeling approaches, we can see that the primary mode captures the change in scale. The secondary mode shows significant changes in the size of the vertebral body and spinous process, especially in the lower thoracic-lumbar regions. The third mode shows a similar change in the vertebral body and spinous process in the lumbar region but the changes are more clearly seen in the MLMO shape model. The particles are in good correspondence and the shape reconstructions are smooth, preserving the anatomical correctness of the vertebrae for the proposed MLMO shape model as compared to the global shape model. The global shape model has inconsistencies in particle correspondence, denoted by jagged shape reconstruction, especially in the posterior arch of the vertebrae, and also weaker correspondence is seen in regions where the lower end of vertebrae comes in close to each other.

**FIGURE 4:**
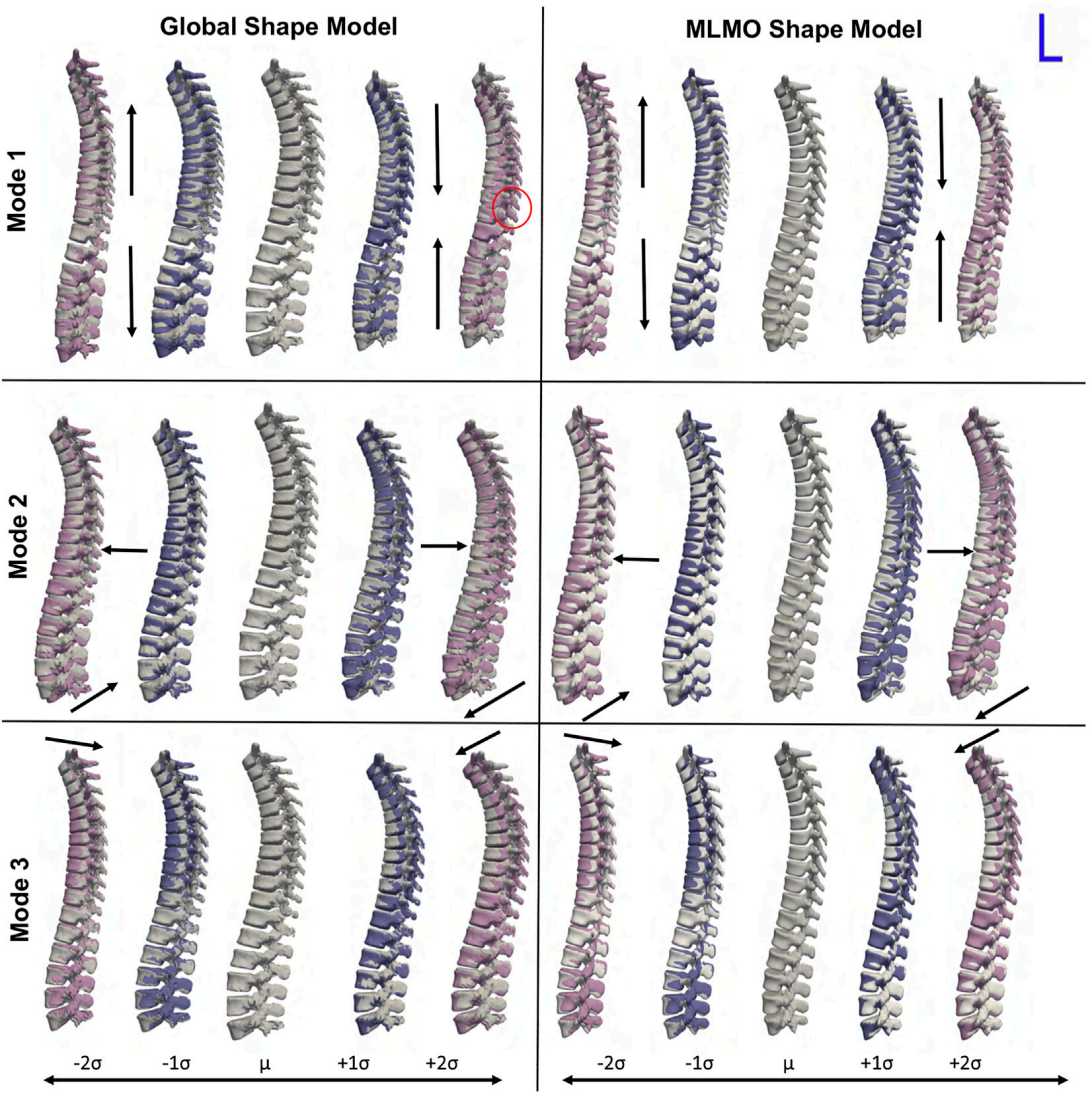
Between-organ modes of variation showing relative pose variations in the spinal column. The mean shape is grey in color with ± 1*σ* modes shown in blue and ± 2*σ* modes shown in pink color. Some of the anatomical inconsistencies are highlighted in red circles and significant pose variations are annotated using arrows.

Figure 4 shows the between-organs mode of variation illustrating the relative alignment variations of the spine. Similar modes were observed for both shape models. The between modes explain the global shape of the spinal curve passing through the vertebral body centers. The primary mode of variation is the elongation and compression of the entire vertebra column which depicts the change in inter-vertebral spaces. However, more penetration of bones is seen in the middle arch of the vertebrae column for the global shape model as compared to the proposed MLMO shape model. The secondary mode captures the curvatures of the thoracolumbar spine segments. It well reproduces the natural variations in the spine when the lumbar segment curvature (lordosis) compensates for the thoracic segment curvature (kyphosis). The third mode captures the variations in the upper-thoracic segment curvature. To see some clinically relevant modes, we can fix one of the vertebrae and observe alignment variations around that vertebra. One such mode is observed when we fix L3 as the origin referential frame, a spine kyphosis variation from hyper-kyphosis (an adult spine deformity pathology where the spine curvature is important), passing to asymptomatic kyphosis for the mean model (moderated natural angle), towards hypo-kyphosis (straight spine, also pathological).

#### 3.2.3 Quantitative results

**FIGURE 5:**
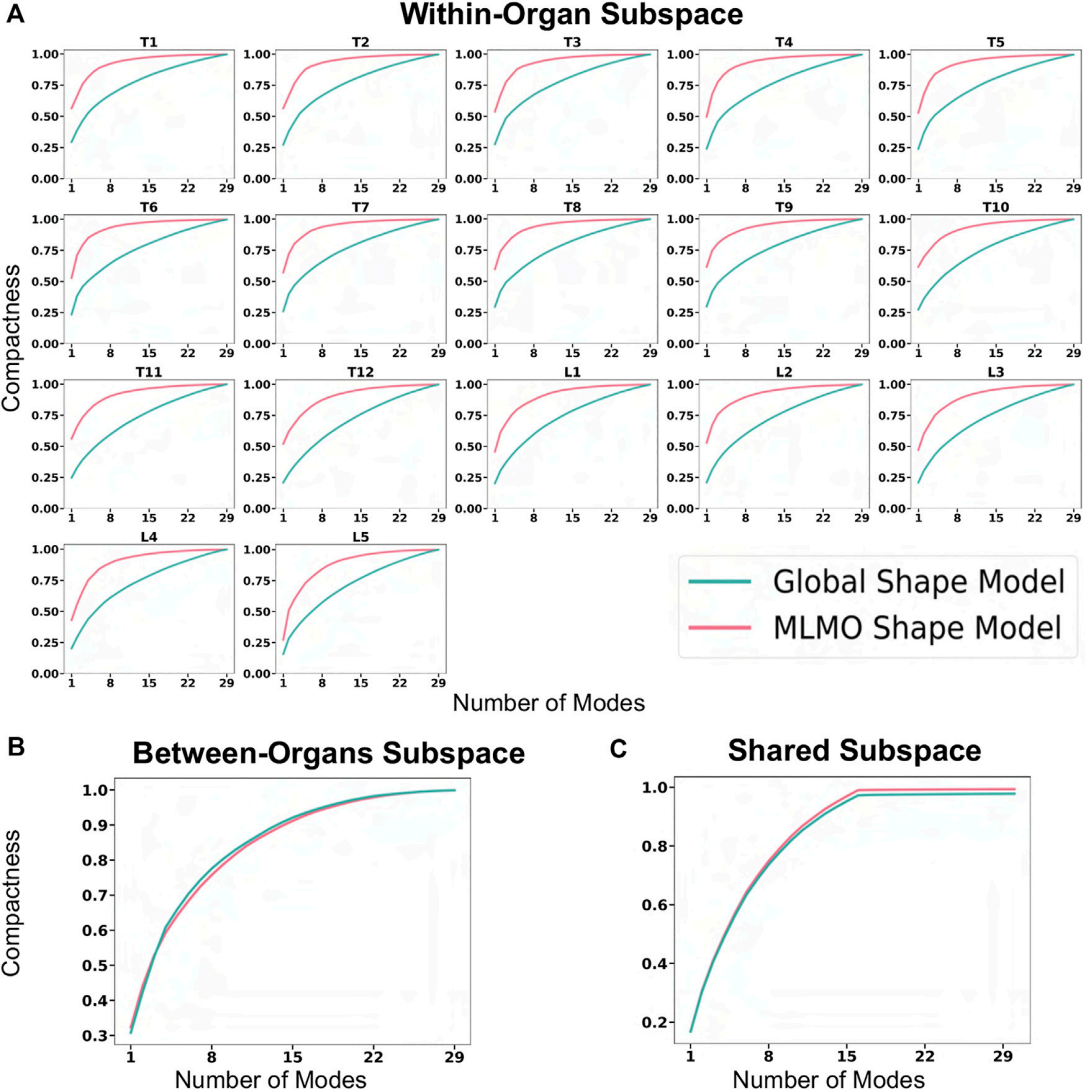
Compactness metric for spinal column data—**(A)** within-organ subspace. **(B)** Between-organs subspace. **(C)** Shared subspace.

The evaluation metrics described in Section 2.6.2 are used to quantitatively assess the proposed shape modeling approach and compare it to the baseline method of the global shape modeling approach. Figure 5A shows the within-organ compactness for each individual vertebra from T1 to L5. We observe that the proposed MLMO shape model gives a compact shape model in the within-organ subspace better than the joint shape model. The shape variations of each vertebra can be explained by less number of modes for the MLMO shape model as compared to the global shape model. To explain 99% of variance, MLMO shape models need 15 modes as compared to more than 20 modes needed by the global shape model. Figures 5B, C show the compactness in the between-organ subspace and in the shared shape space and we can see that the compactness measure is nearly the same for both the approaches.

**FIGURE 6:**
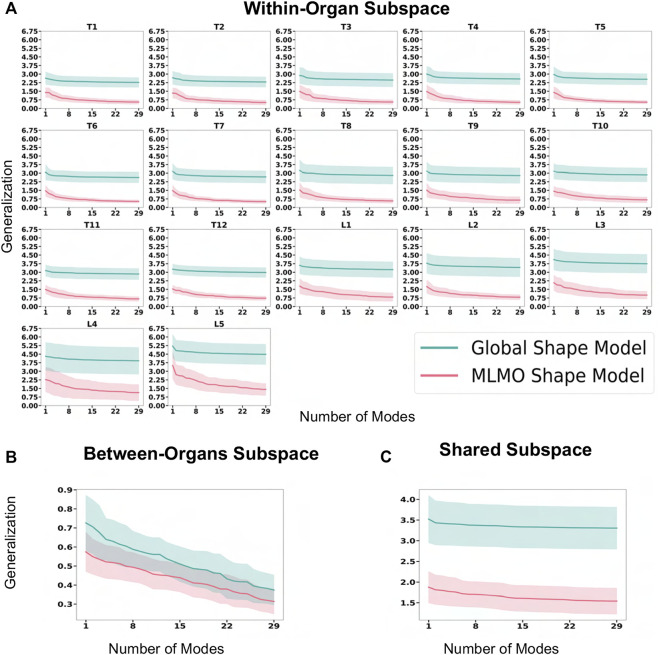
Generalization error (in *mm*) for spinal column data—**(A)** within-organ subspace. **(B)** Between-organs subspace. **(C)** Shared subspace.

**FIGURE 7:**
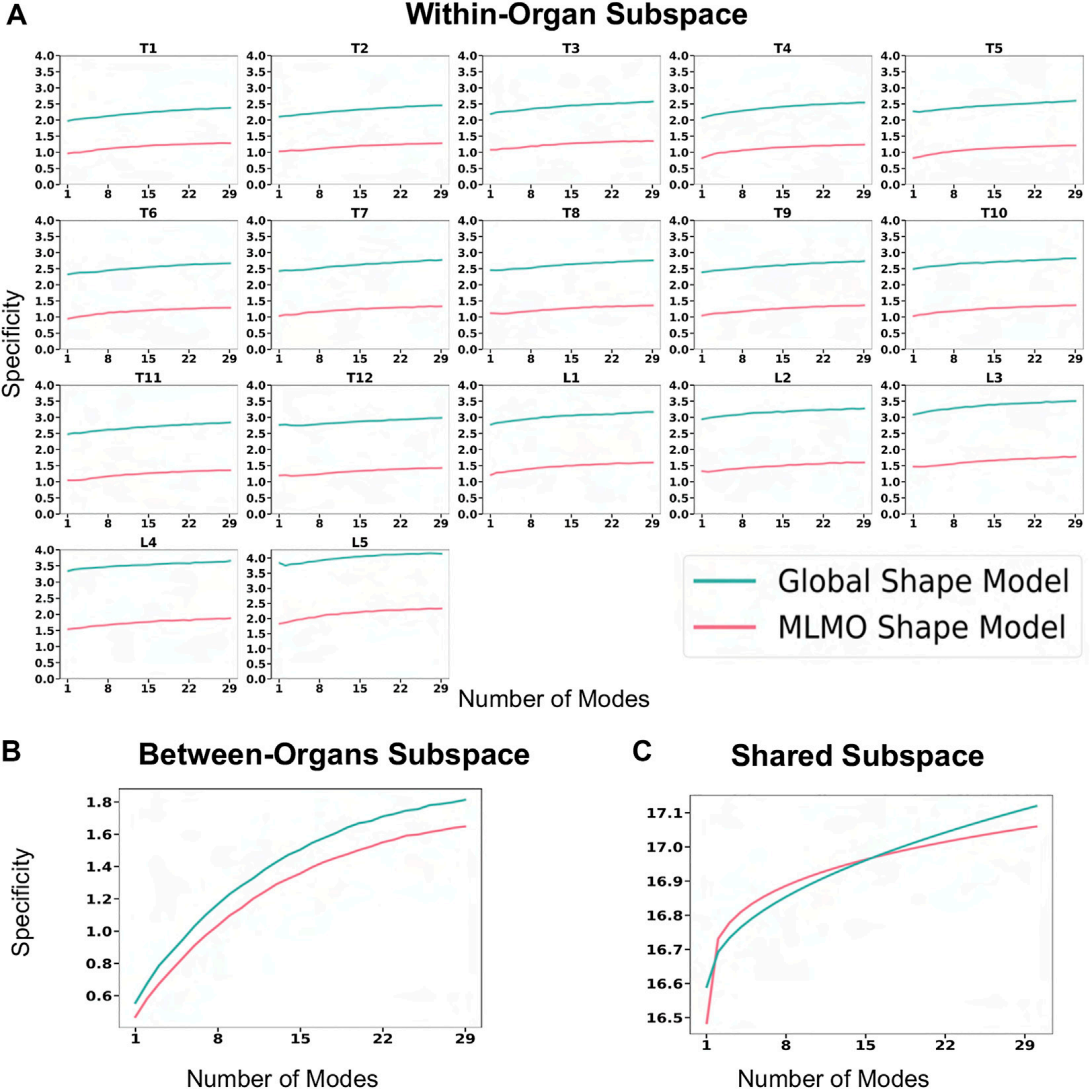
Specificity (in *mm*) for spinal column data—**(A)** within-organ subspace. **(B)** Between-organs subspace. **(C)** Shared subspace.

Figure 6A shows the within-organ generalization error for each individual vertebra in the multi-organ structure. On average, the MLMO shape model has a generalization error of 1.5 mm and 2.5 mm in the thoracic and lumbar regions, whereas, the global shape model has a generalization error of 3.0 mm and 4.5 mm for the thoracic and lumbar regions, respectively. MLMO shape model consistently improves the generalization on the held-out samples for each vertebra as compared to the global shape model. This implies that the shape morphological variations of each bone are generalized well by the proposed shape modeling technique. Figure 6B shows the generalization error in the between-organs subspace and we can see that the proposed MLMO approach can generalize well for unseen relative alignment of the bones in the multi-organ model of the vertebra as compared to the global approach where the relative pose is not optimized during the shape modeling. Thus, it can be seen that by optimizing the shape and relative pose subspace separately, we can get a shape model which can generalize well to unseen morphological changes of each vertebra and also to their unseen relative alignment. From Figure 6C, it can be seen that the proposed modeling approach gives a lower generalization error in the shared PCA subspace. Figure 7 shows the specificity measures in different subspaces. The MLMO shape model is more specific in the shared, within, and between subspaces.

#### 3.2.4 Validation results

To investigate the relevance of our proposed shape modeling approach, we experimented to use the resulting shape descriptors as a predictor in a regression task. Therefore, an experiment is formulated to compare the predicted patient age by the shape model versus the ground-truth age. Correspondence particles of a statistical shape model have the potential to produce additional diagnostic, predictive, and prognostic information beyond what is visually perceptive and hence can be used for various downstream tasks which are clinically relevant Goparaju et al. (2018). Several clinical studies have shown age-related morphological and alignment changes in the vertebra. Osteophyte formation involves an increase in the vertebral endplate dimensions, and activity-induced lifelong periosteal growth Junno et al. (2015) and Whitmarsh et al. (2012) or due to some other underlying pathological condition. This validation task aims to study which shape modeling technique’s correspondence particles are more predictive of the patient’s age and is based on the hypothesis that due to the temporal features of age progression, the correspondence particles generated by the shape model can also display a sequential pattern of low-dimensional distribution of age progression. With the age regression on the shape descriptors, we aim to corroborate that the proposed model efficiently captures the morphological changes related to normal aging evolution. These aging variations of the spinal column are typically related to the narrowing of the spinal canal, increase in endplate size and convexity, decrease in vertebral body height, and increase in pedicle diameters Whitmarsh et al. (2012). The idea is to compare both model regressions (MLMO and global shape Model) to see if the MLMO shape model has more prediction power (better *R* ^2^ metric). A regression model is built using the correspondence particles generated by the shape model to predict the age of the same shape cohort of 30 subjects as described in Section 3.2 having mean age of 60 ± 17 years. We applied random sampling and selected 30% of subjects to be used as a testing dataset which is held out from the initial analysis. Feature vectors for the regression model were generated by projecting the correspondence particles to the shared PCA subspace for the global shape modeling approach and the MLCA subspace for the proposed MLMO shape modeling approach. For both approaches, we select features up to the number of modes that can explain 97% variability across the population. We used Least Absolute Shrinkage and Selection Operator (LASSO) as the regression model for our experiment Friedman et al. (2010). The independent variable is the subject’s age and the dependent variables are the correspondence particles of the training set which are the shape descriptors in the PCA and MLCA subspaces, respectively for the two shape models. The coefficient of determination *R* ^2^ is computed to assess the two trained regression models. This metric is related to the regression residuals and is defined as:

$$R^{2}=1-\frac{\sum_{i=1}^{T}{\left(y_{i}-\tilde{y_{i}}\right)}^{2}}{\sum_{i=1}^{T}{\left(y_{i}-\bar{y}\right)}^{2}}$$ (12)

where *T* is the number of test subjects, *y*_*i*_ is the actual patient age and $\tilde{y_{i}}$ is the patient age predicted from the shape model and $\bar{y}$ is the mean patient age. The regression model was tuned using a five-fold cross-validation approach to get optimal regularization weight for the curve fit. The *R* ^2^ metric was then calculated on the testing dataset. The age regression curve fitted for the MLMO shape modeling approach has an *R* ^2^ value of 0.62 with a mean predicted age of 63 ± 8 years and for the global shape modeling approach, the *R* ^2^ value is 0.20 with a mean predicted age of 46 ± 15 years. As we have a drawback of having a small number of samples in the regression model, we studied the influence of sample number on the regression model. We repeated this experiment by training it on a specific percentage of subjects coming from the training data and then increasing the percentage of training shapes. The *R* ^2^ metric values coming from these experiments are then interpolated using a power law curve. Under the power law curve assumption, we should see a significant improvement in accuracy if we increase the training data size. This helps in getting an estimate of the evaluation metric value at a point when we have a sufficient number of training shapes available. The results from this experiment are shown in Figure 14A and we observe that there is an improvement in the *R* ^2^ score for both the shape models. The *R* ^2^ value for the regression model built for the MLMO shape model increases to 0.81 and the *R* ^2^ score increases up to 0.31 for the global shape model if we have a training dataset of size 1.2 times the current size. These results suggest that the correspondence particles generated from the MLMO shape modeling approach are more predictive in capturing the morphological and relative alignment variations of the vertebra column which arise due to changes in age. We hypothesize that as the proposed MLMO shape model optimizes particle correspondences separately on the within-organ and between-organs subspaces, it can better capture the feature changes of the vertebra both morphology as well as configuration-wise. However, this validation task has certain limitations that warrant consideration. The results are shown on a very small dataset and the gender of the subjects is not taken into consideration. However, there seems to be a correlation between gender and vertebral pathology, which limits the generalizability of the validation results.

### 3.3 Foot and ankle

#### 3.3.1 Dataset

Dataset comprising images from weightbearing CT (WBCT) scans (Planmed Verity - 0.4 × 0.4 × 0.4 mm voxels) for the foot and ankle of twenty-seven asymptomatic participants is used to build shape models. The subjects’ age was in the range of 50.0 ± 7.3 years, with height and BMI in the range of 169.4 ± 6.4 cm and 25.3 ± 3.8 *kg*/*m* ^2^, respectively. The bones of interest (namely, the calcaneus, talus, navicular, and cuboid), make up the hindfoot and part of the midfoot, which is comprised of the subtalar, talonavicular, and calcaneocuboid joints. Due to the complex morphology and joint relationships within these four bones, current 2D radiographic measurements fail to quantify the 3D morphology and joint relationships properly. Computationally modeling these morphologies and joint relations could yield increased clinical understanding of pathologies, improved surgical planning, and advanced implant design. The WBCT scans were subjected to segmentation, decimation, and smoothing to generate 3D surface models of the talus, calcaneus, navicular, and cuboid. We build shape models using 1,024 correspondences for the talus, 2048 for the calcaneus, and 512 for both the navicular and cuboid bones.

#### 3.3.2 Qualitative results

**FIGURE 8:**
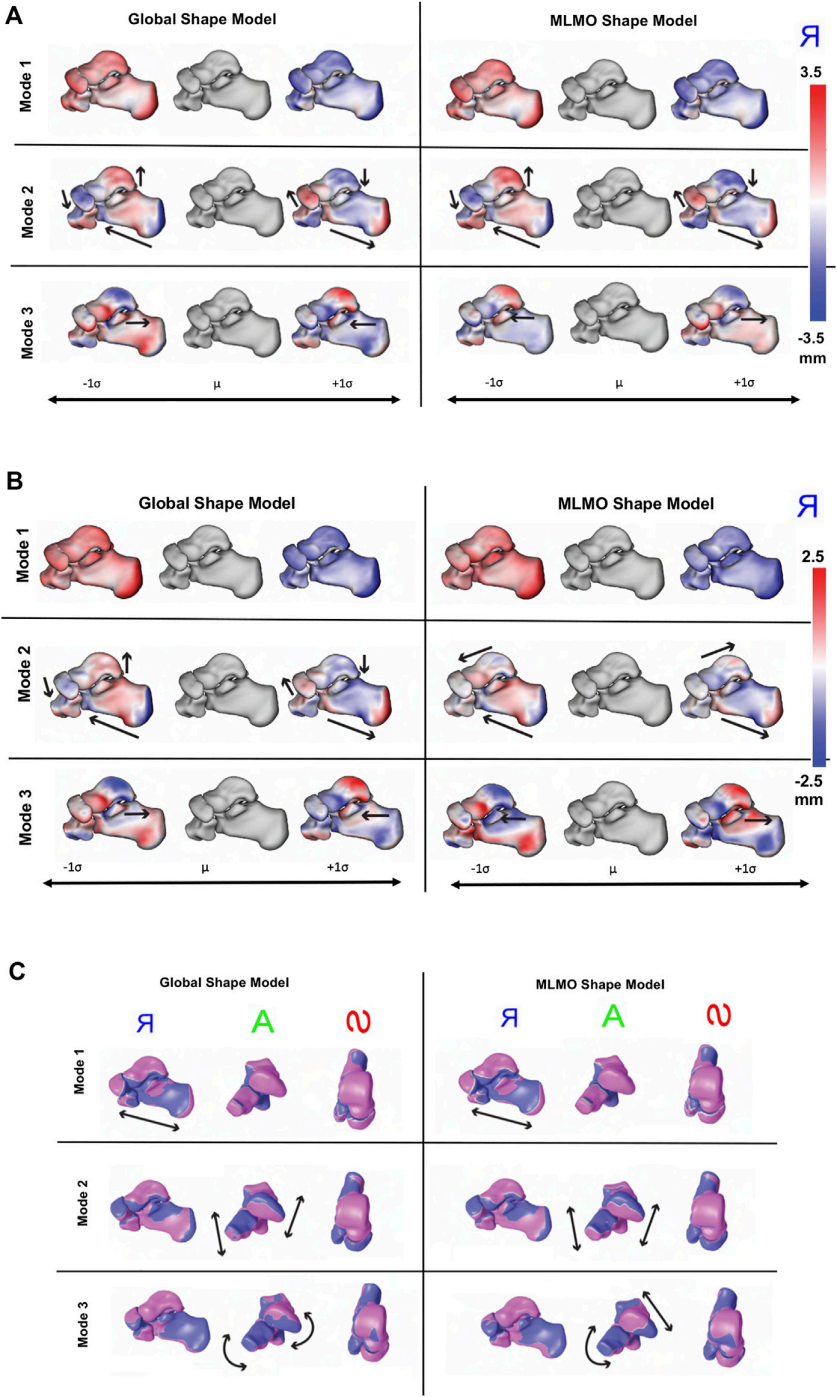
Qualitative results for foot and ankle data. **(A)** PCA modes of variation. The color map shows the distance of each mode from the mean shape. mean shape. The arrows denote the direction along which significant shape changes take place. **(B)** Within-organ modes of variation. The color map shows the distance of each mode from the mean shape. The arrows denote the direction along which significant shape changes take place. **(C)** Between-organs modes of variation—Medial, Anterior and Superior View. The mean shape is grey in color with +1 σ modes shown in blue and −1 σ modes shown in pink color.

Figure 8A shows the modes of variation for PCA in the shared subspace of all the bones. Both the shape modeling techniques give similar morphological and configurational modes while maintaining the joint articular relationships. The primary mode highlights the overall growth and shrinkage of all four bones simultaneously. The secondary PCA modes remain entangled in terms of alignment and morphology and there is an inverse relationship between the calcaneus and talus. As the calcaneus lengthens, the posterior facet’s slope decreases and when the two bones are analyzed together simultaneously, we see that when the talar dome heightens, the posterior process diminishes and the calcaneus shortens with the posterior facet’s slope increasing. Moreover, as the talar dome heightens, we see that the navicular and cuboid slide inferiorly with very less rotation around the anterior-posterior axis. The third PCA mode shows variation in the anteromedial facet such that it moves from the anterior to posterior direction as we move along different standard deviations of modes.

Figure 8B shows the within-organ modes of variation. We observe identical morphological modes for both shape modeling techniques. The primary mode here as well highlights the change in scale and shows each bone increasing and shrinking in size individually. The secondary mode shows the lengthening of the calcaneus with a simultaneously decreasing posterior facet slope. We can still observe modest talar dome, navicular and cuboid changes, but they are not as dominant as the PCA modes discussed above. The third mode emphasizes a similar anterior/posterior anteromedial facet variation but this is accompanied by a rotational component. We also see that the anteromedial facet’s slope changes as we move along the standard deviations from a steep slope to a more flattened slope.

Figure 8C shows the between-organ modes of variation. We notice similar modes for both shape modeling techniques, highlighting significant variations in the overall configuration of the articulated joint while preserving the mean morphology, which was not seen directly in the PCA modes. The primary mode of variation is an overall outward and inward movement between the bones which effectively increases and decreases the joint space distance. The secondary mode primarily emphasizes the superior and inferior motion of the four bones such that as the talus moves along the superior direction, the calcaneus, cuboid, and navicular move along the inferior direction. The third mode reflects the medial, and lateral movement of the talus and calcaneus such that as the talus moves along the medial axis, the calcaneus moves laterally. Moreover, we see as the talus moves in the medial direction, the navicular rotates along the superior and lateral axis, and the cuboid rotates in an inferior and medial direction.

#### 3.3.3 Quantitative results

**FIGURE 9:**
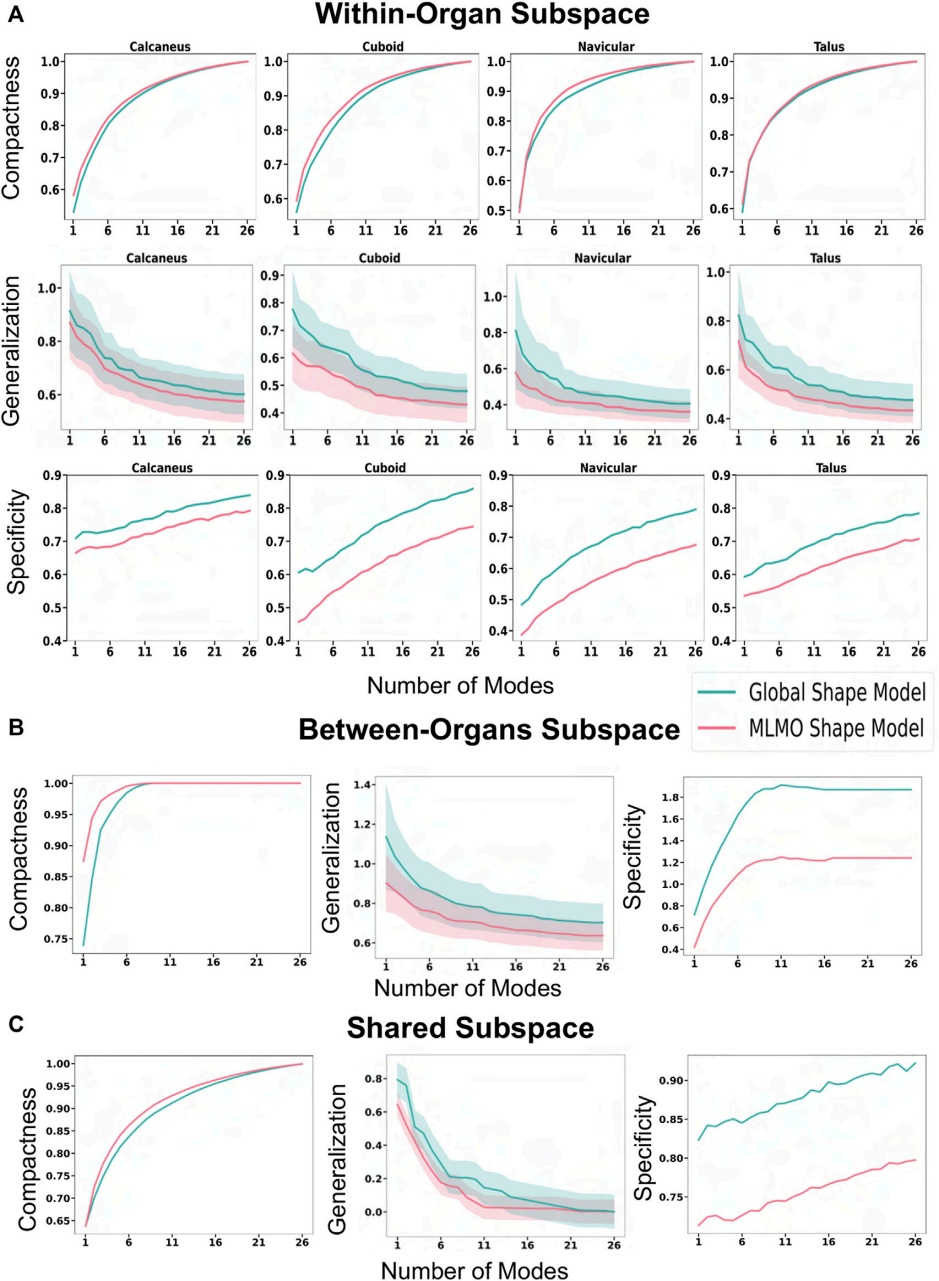
Quantitative Evaluation metrics (compactness, generalization (in *mm*), and specificity (in *mm*)) for the foot and ankle dataset in **(A)** within-organ **(B)** between-organs **(C)** shared subspaces.

Figure 9 shows the quantitative evaluation metric results. The compactness measure for the MLMO shape model is higher as compared to the global shape model, although the difference is not very pronounced. The MLMO shape model gives lower generalization and specificity errors in each subspace (within, between, shared), which indicates that it can generalize well to unseen morphological and pose variations of the ankle joint, either combined or separately.

#### 3.3.4 Validation results

Joint level measurements serve as an important tool to better understand the joint level morphology and alignment variations and to improve ankle joint pathological diagnosis and operative procedures. To validate the proposed shape modeling technique, we used the shape model to predict the joint coverage area of the articulating region of the subtalar joint which is the joint between two of the tarsal bones (the talus and calcaneus) in the foot. Coverage area can be used to gain useful insight and quantify the morphological variations, like osteoarthritis development and alignment variation, such as joint subluxation Schaefer et al. (2012) and Louie et al. (2014).

Samples from the entire dataset were randomly sampled into train-test splits with seventy percent of samples selected for the training of the shape models using the proposed and the global shape modeling approach. Each test shape sample is then orthogonally projected onto the PCA subspace for the global shape modeling approach and onto the within-organ and between-organs subspaces for the proposed MLMO shape modeling approach, and then reconstructed back following the generative equations of PCA and MLCA as described in Eqs 4, 7. We then compare the coverage of the subtalar joint for the reconstructed sample to the ground truth coverage measurements of that subject. To calculate the coverage area between two bones we use normal vectors from each face of one of the bones and identify which faces those vectors intersect with on the opposing bone. We consider that normal vector to be within coverage only if it intersects with an opposing face and the surface area was calculated on that identified region. To measure the error in coverage area between the ground-truth and reconstructed shape samples, we use relative error *ϵ* as an evaluation metric which is defined as

$$\epsilon =\frac{\left|\tilde{a}-a\right|}{a}$$ (13)

where $\tilde{a}$ is the predicted coverage area of the subtalar joint measured from the reconstructed shape complex and *a* is the coverage area of the subtalar joint for that particular subject computed on ground-truth meshes. We repeated this experiment using five-fold cross-validation. Figure 14B shows the box plot for the relative errors. We can see that the relative error for the proposed MLMO shape model is comparatively smaller than the global shape model, although this difference is small. The mean relative coverage area error of calcaneus and talus is 4.0% and 3.6%, respectively, for the proposed MLMO shape modeling approach. For the global shape modeling approach, the errors are 4.4% and 3.8% for the calcaneus and talus, respectively. In Table 1, we report the population level coverage area measurements of the subtalar joint for the five-fold cross-validation experiments from each of the optimization approaches along with the ground truth coverage measurements. These results suggest that the MLMO shape modeling technique is better at preserving the true anatomical correctness of the articulated joint (indicated here by coverage area) while simultaneously building a compact model.

**TABLE 1:** Coverage area measurements (*μ* ± *σ* in *mm* ^2^) of the subtalar joint.

	Talus	Calcaneus
Ground truth	1527.87 ± 201.30	1439.94 ± 186.78
Global shape model	1503.08 ± 164.38	1413.19 ± 141.92
MLMO shape model	1481.22 ± 165.38	1391.91 ± 140.33

### 3.4 Hip joint

#### 3.4.1 Dataset

We used a dataset of 51 hemi-pelvis and proximal femur pairs of the hip joint. The shape cohort comprised of 24 control subjects, six patients with cam femoroacetabular impingement (FAI), 10 patients with acetabular dysplasia and 10 patients with pelvic retroversion. The shape models were built using 2048 correspondences on the pelvis and 512 on the femur, using the two shape modeling approaches.

#### 3.4.2 Qualitative results

**FIGURE 10:**
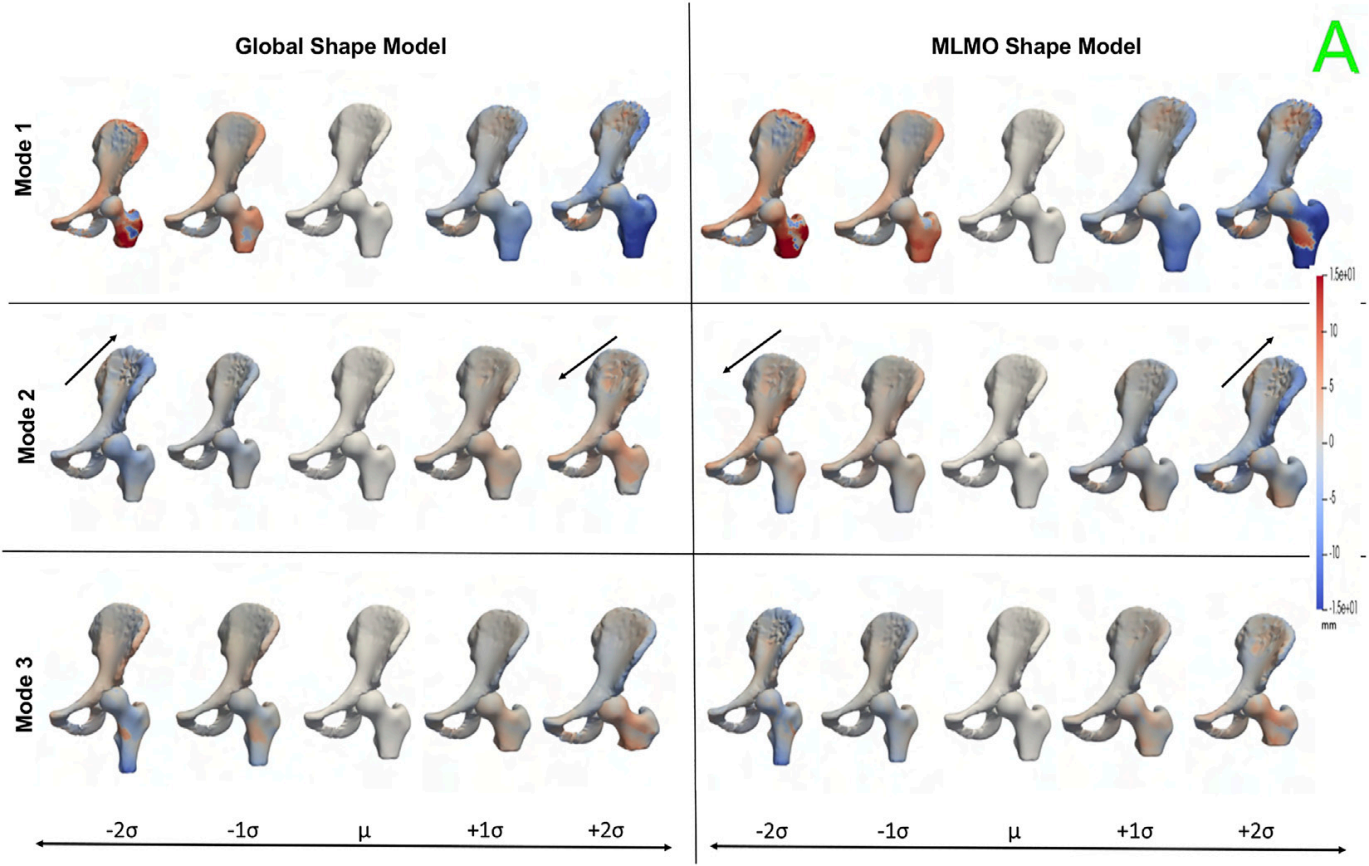
PCA modes of variation in the shared subspace for the hip joint dataset. The color map shows the distance of each mode from the mean shape. The arrows denote the direction along which major shape variations take place.

Figure 10 shows the PCA modes of variation in the shared subspace. We observe similar modes for both the shape models with a difference in magnitude, with the global shape model having reduced magnitude of variation. The primary mode highlights the shape variation associated with overall growth and shrinkage in size, along with an asphericity of the femoral head likely attributed to some of the subjects having cam FAI morphology. The second mode highlights anterior-posterior pelvic tilt entangled with shape variations on the femoral head and the ilium. The third mode shows elongation and shortening of the femoral shaft attributed to the field of view of the imaging, as well as changes in the curvature of the ilium.

**FIGURE 11:**
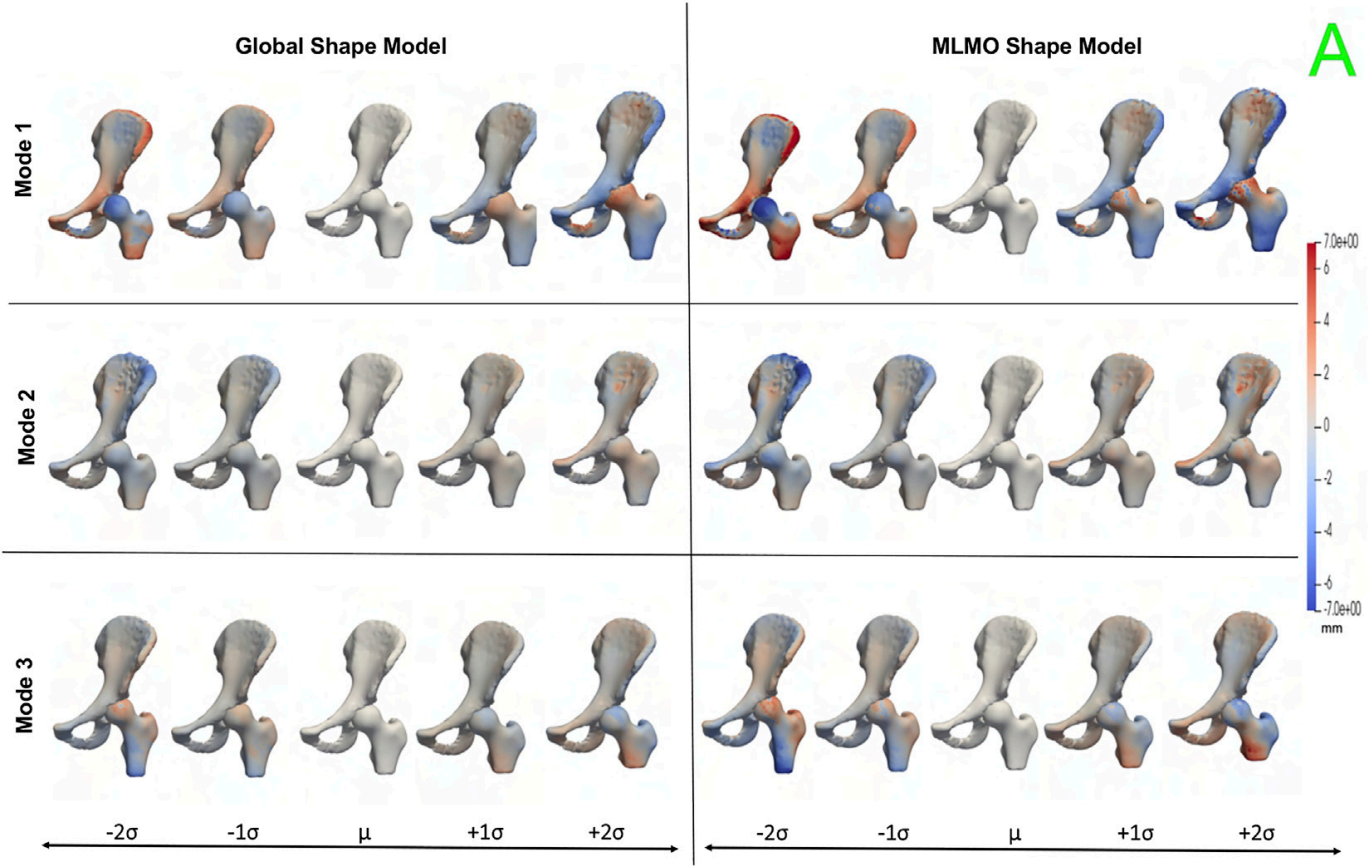
Within-organ modes of variation showing morphological changes in each bone for the hip joint dataset. The color map shows the distance of each mode from the mean shape.

Figure 11 shows the within-organ modes of variation highlighting morphological changes of pelvis and hip. The primary mode shows the changes in scale as growth and shrinkage variations. There are some morphological changes on the femoral head as well. The secondary mode shows the morphological changes of the ilium with minimal shape variation for femur. The third mode shows the shape variations in femoral head and shaft. The shape variation capture by the modes of the proposed MLMO shape model are of higher magnitude as compared to the global shape model.

**FIGURE 12:**
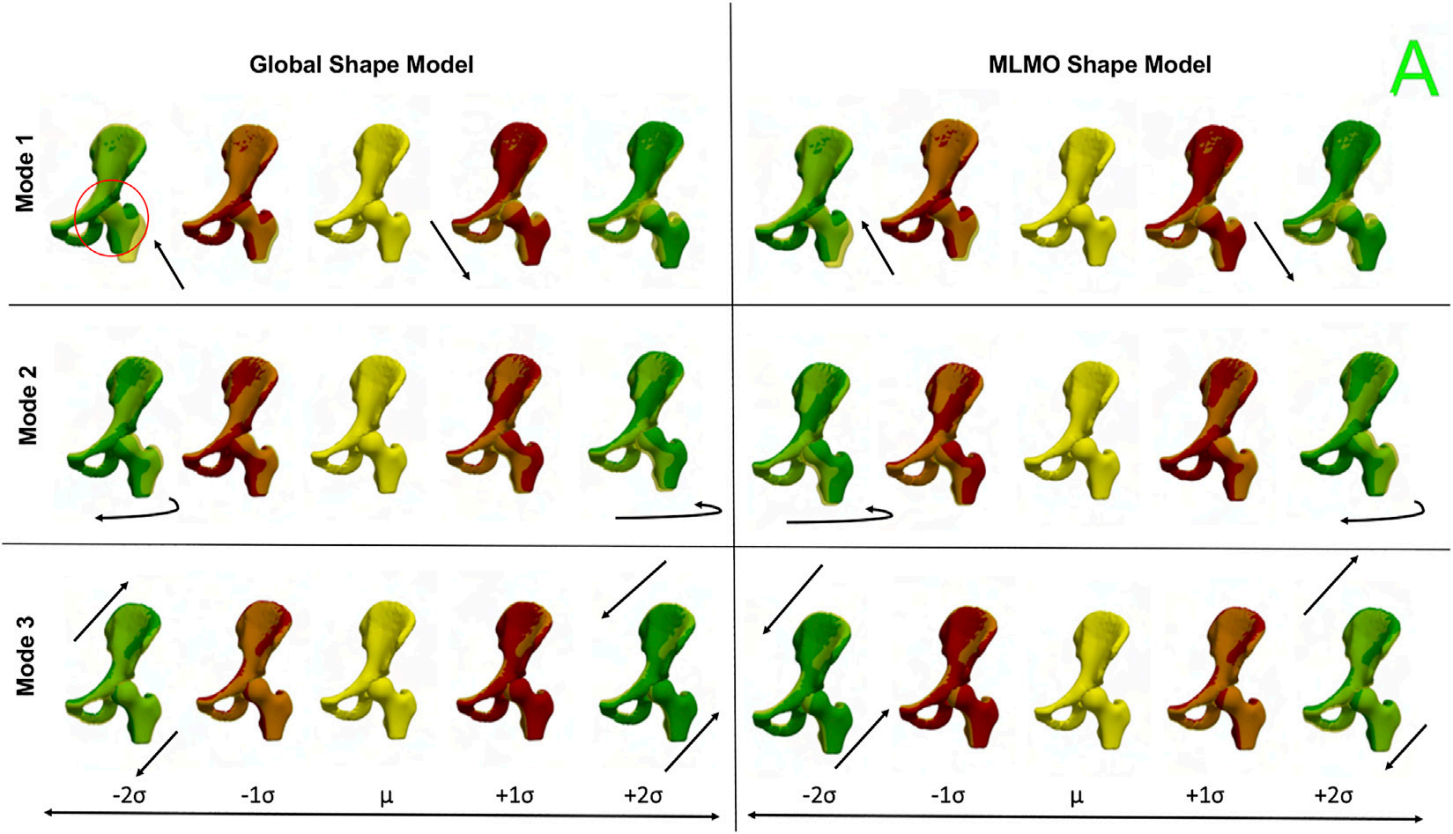
Between-organs modes of variation showing relative pose variations for the hip joint dataset. The mean shape is yellow in color with ± 1*σ* modes shown in red and ± 2*σ* modes shown in green color. The arrows denote the direction along which significant pose variations take place.

Figure 12 shows the between-organ modes of variation depicting the relative alignment variations of the hip joint while simultaneously preserving the mean shape. In the primary mode, we observe the increased and decreased space between the femoral head and the acetabulum. This observation may be unique to this dataset, as the images were acquired with the hip in traction to widen the intra-articular joint space for visualization of the separated cartilage layers during CT image acquisition. The variability in the amount of traction applied cannot be factored out by the initial rigid alignment process. However, we notice that the non-physiological penetration of femoral head into the acetabulum is more pronounced in the modes discovered by the global shape model. The second mode shows the femoral head tilting towards the posterior direction with the pelvis fixed, representing variation in flexion-extension of the hip joint. The third mode shows minor alignment shift between the pelvis and femur in the opposite direction, such that when pelvis shifts laterally the femur shifts medially and vice versa.

#### 3.4.3 Quantitative results

**FIGURE 13:**
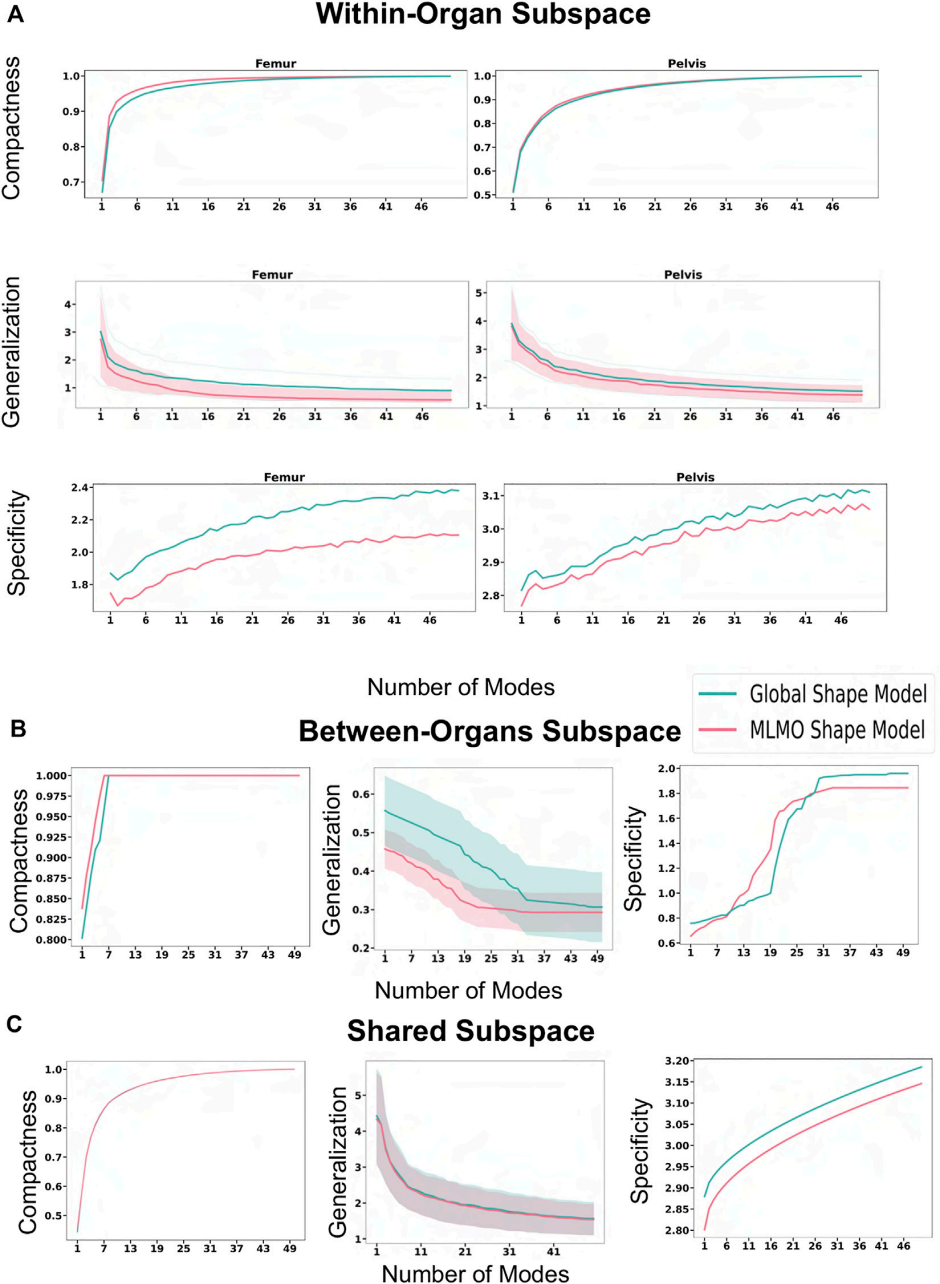
Quantitative Evaluation metrics (compactness, generalization (in *mm*), and specificity (in *mm*)) for the hip joint dataset computed in **(A)** within-organ **(B)** between-organs **(C)** shared subspaces.

Figure 13 shows the quantitative evaluation metric results. The compactness measure for the shape models, both baseline and proposed are very close to each other. The MLMO shape model has lower generalization and specificity errors as compared to the global shape model in the between and within subspaces but has similar errors in the shared subspace. The specificity measure is lower for the MLMO shape model in each subspace. In the within subspace, the generalization and specificity metrics are higher for the pelvis than the femur for both shape models.

#### 3.4.4 Validation results

**FIGURE 14:**
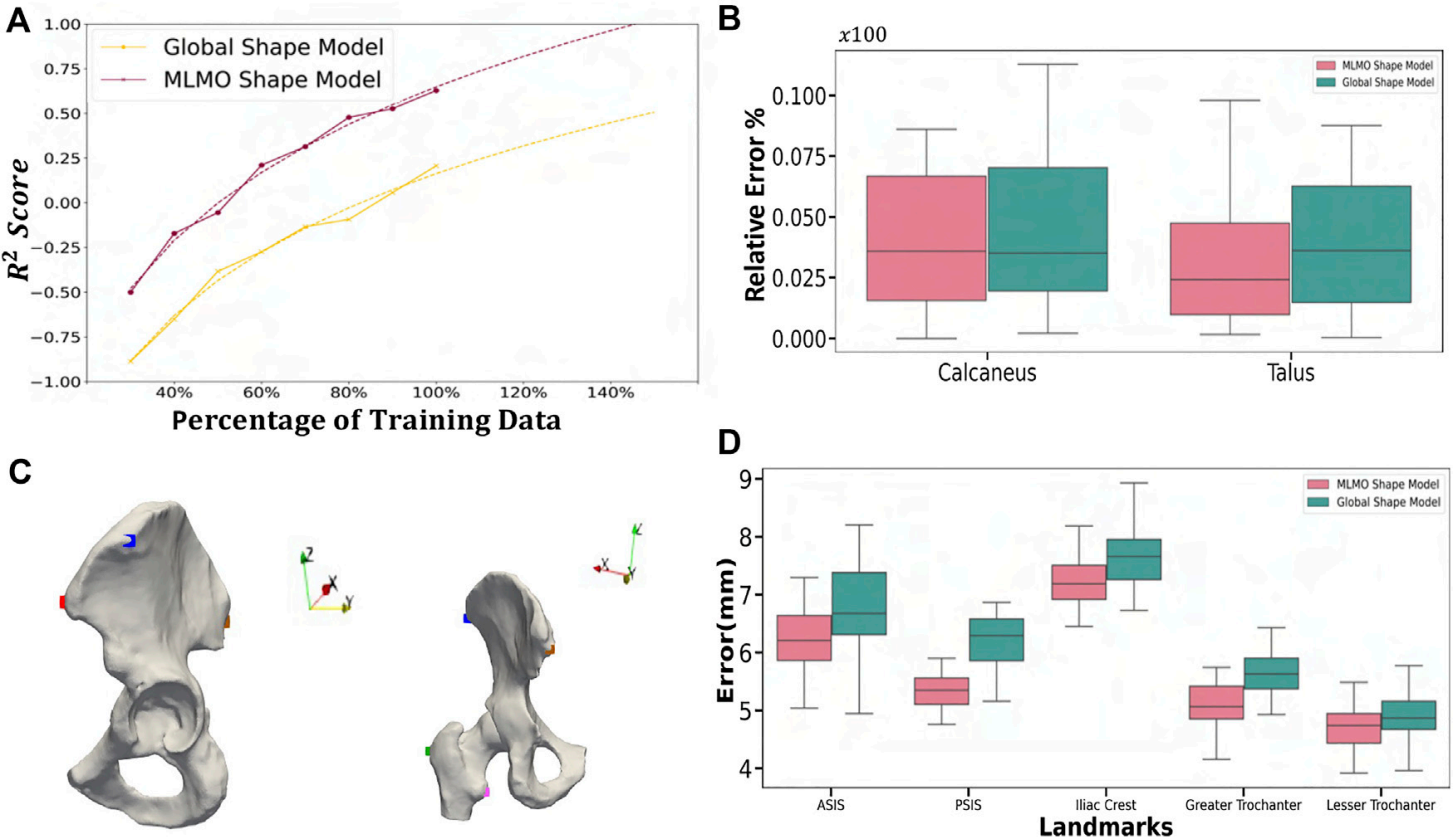
Validation Results—**(A)** Power law interpolation for *R* ^2^ metric computed at different training data sizes of spinal column data. **(B)** Box-plot for the relative error of coverage area for calcaneus and talus. **(C)** Anatomical landmarks for femur and pelvis used for landmark inference - iliac crest (blue), ASIS (red), PSIS (brown), lesser trochanter (pink) and greater trochanter (green). **(D)** Box-plot for Euclidean distance error computed for the landmark inference validation task done on the hip joint.

Statistical shape models can be employed to automate the inference of patient-specific anatomical morphometrics. For the validation task using the hip joint data, we estimated the patient-specific anatomical landmarks for the pelvis and femur anatomies. The estimation of landmarks is an important task as landmarks are used for a variety of clinical and research applications, like motion tracking or coordinate system identification for surgical planning or robotic surgery. The given dataset was randomly sampled into train-test split with 30% of subjects held out as testing dataset. The shape model was generated on the training dataset using the baseline and proposed methodology. Ground-truth landmarks were manually annotated by an expert using first and second principal curvature of the surfaces for guidance. We chose five landmarks for the hip joint in our validation task as shown in Figure 14C. Three landmarks were defined on the pelvis, including the anterior superior iliac spine (ASIS), posterior superior iliac spine (PSIS) and iliac crest, as are commonly used in motion capture and for the development of a pelvic coordinate system. Two landmarks were defined on the femur, including the greater trochanter and lesser trochanter. The point correspondences for each test subject were then generated using the shape model of the training data. The subject-specific landmarks were then warped from the subject space to the mean space of the shape model using thin plate splines (TPS) Bookstein (1989). This was followed by constructing a TPS warp using correspondences of the mean shape and the subject-specific anatomy as reference points, which served as a mapping between the mean and subject spaces. Finally, the mean landmarks were warped back to subject space to obtain subject-specific landmarks which were the predicted points from the SSM. We then computed the Euclidean distances between the predicted landmarks and the ground-truth values. We repeated this process five times on different train-test splits to get five-fold cross-validation metrics for the euclidean distance error. Figure 14D shows the box-plot for the Euclidean distance error in mm for the proposed and baseline methods. The landmarks predicted by the proposed MLMO shape model had comparatively lower errors as compared the ones predicted by global shape model. The errors for the landmarks placed on femur (greater and lesser trochanter) are lower in magnitude as compared to the errors for landmarks on pelvis. The highest errors were observed for the iliac crest landmark, which was placed on the most prominent feature of the iliac crest, a location which is variable across subjects and difficult to identify through palpation of bony prominences for motion capture. From these results, we infer that due to the disentangled approach to build the shape model, the proposed MLMO shape model had particle correspondences which reflected the true morphology of the individual bone (femur or pelvis) which were anatomically correct. A promising direction for the future application of the MLMO shape model motivated by this validation experiment is to calculate joint angle measurements in a relevant clinical/anatomical coordinate system from the surface reconstructions provided by the MLMO shape models. With the MLMO shape model, landmarks that are necessary to define a coordinate system could be based on their spatial relationship to the underlying correspondence particles, which can then be used to calculate pose automatically in a clinically relevant anatomic coordinate system. This work can improve the clinical interpretation of articulated joint data from statistical shape models. We also see the potential of tying the correspondence model to associated biomechanics measurements to quantify form-function relationships. For instance, we can show that patients with more severe cases of hip dysplasia (which can be measured by MLMO) also exhibit more pronounced hip instability (as measured by motion capture).

## 4 Conclusion

In this paper, we proposed a new shape modeling approach for multi-organ anatomies by separating shape from pose and building a shape model by optimizing the mutually orthogonal subspaces of each organ and their relative pose. The proposed method efficiently uses the available, typically limited, 3D models of anatomy to capture subtle, clinically relevant morphological intra- and inter-structural correlations. The method also provides a scalable approach for modeling anatomies with more organs compared to the current global shape modeling scheme that can get prohibitive with increased number of correspondence points and more organs. This offers a practical solution for a wider range of problems in the multi-organ shape acquisition and modeling relative to the work in the literature. We showed from experiments that the MLMO shape modeling technique outperforms the global PSM method by accurately capturing the morphological and configuration variations for multi-object structures. Our model mitigates the problem of overestimation of variance which is the case with global shape models, where PCA in the shared shape space leads to anatomical inconsistencies. The proposed MLMO modeling technique is scalable as the generative model is built individually for each organ and also for their relative pose, thereby leading to covariance matrices of much lower dimension than the joint models. The shape models generated by the proposed PSM method are more compact, specific, and generalizable as compared to the global shape models in high dimensional, low sample size settings. Due to the generative nature of the proposed MLMO shape modeling technique, this method is orthogonal to and can be extended to various posterior inference techniques that are applied to traditional statistical shape models Albrecht et al. (2013). An intriguing direction for a future work application using the MLMO shape modeling technique could be to infer the shape and pose relations when some organ shapes are known a priori, and the objective is to model the posterior distribution of the entire multi-organ shape given the known partial parts. The additional benefit of learning conditional distribution using MLMO shape models for articulated joints will be that it can be used to reconstruct and understand the healthy morphology of the shape with simultaneously restoring the native joint biomechanics as the shape and relative pose subspaces remain disentangled in the proposed approach.

Our work comes with some limitations. The proposed MLMO shape modeling technique builds from the idea that configuration variations in the pose can be learned by modeling the distance of the centroid of each object from the global centroid. This enables us to disentangle the shape from its relative pose and gives us a simple way to learn the relative alignment of structures in the multi-organ shape complex, along with the morphological changes in each object. Although this linear assumption that a Gaussian distribution can model each subspace helps us to bring anatomical correlations in terms of relative pose between joint structures in multi-organ settings to the shape modeling process, however, these relative pose variations cannot be entirely linear, and might have some non-linear variations across the shape and pose features. A direction for future work can be to incorporate a more robust generative model that can learn linear and non-linear embeddings of the high-dimensional shape and pose spaces in low-dimensional space in a fully probabilistic manner for multi-organ anatomies. This will improve the capability of the multi-organ shape models to handle complex inter-object pose relations better to build shape models of complex anatomies as realistically as possible.

## Data Availability

The raw data supporting the conclusion of this article will be made available by the authors, without undue reservation.

## Footnotes

1
*d* = 3 in this work, however PSM and the proposed approach is flexible to model any dimension.
